# Autosomal dominant gain-of-function mutations in LCP1 cause a syndromic neutropenia and immunodeficiency

**DOI:** 10.1016/j.gendis.2026.102232

**Published:** 2026-05-12

**Authors:** Lang Yu, Bo Zhou, Wei Liu, Wenhui Li, Qinglv Wei, Yishi Zhang, Chen Li, Wendao Li, Yulin Li, Guangzhao Li, Gan Sun, Rui Gan, Ran Chen, Wenjing Zhang, Anle Zeng, Rongtao Zhao, Wenli He, Yanjun Jia, Lina Zhou, Zhiyong Zhang, Xuemei Tang, Xiang Qiu, Qing Zhou, Wenxia Song, Xiaodong Zhao, Yunfei An

**Affiliations:** a National Clinical Research Center for Child Health and Disorders, Ministry of Education Key Laboratory of Child Development and Disorders, Children's Hospital of Chongqing Medical University, Chongqing 400014, China; b Chongqing Key Laboratory of Child Rare Diseases in Infection and Immunity, Children's Hospital of Chongqing Medical University, Chongqing 400014, China; c Department of Biology, Southern University of Science and Technology, Shenzhen, Guangdong 518055, China; d Department of Hematology and Oncology, Children's Hospital Affiliated to Zhengzhou University, Zhengzhou, Henan 450018, China; e Department of Rheumatology & Immunology, Children's Hospital of Chongqing Medical University, Chongqing 400014, China; f Life Sciences Institute, Zhejiang University, Hangzhou, Zhejiang 310058, China; g Liangzhu Laboratory, Zhejiang University, Hangzhou, Zhejiang 311121, China; h Department of Cell Biology and Molecular Genetics, University of Maryland, College Park, MD 20782, United States

**Keywords:** Immuno-actinopathy, Immunodeficiency, Inborn errors of immunity, LCP1, Neutropenia

## Abstract

Actin cytoskeleton defects underlie immuno-actinopathies. We identified three patients with heterozygous LCP1 gain-of-function mutations (L362F, A365D) causing activated LCP1-associated immunodeficiency syndrome, characterized by congenital neutropenia, variable combined immunodeficiency, and allergy. Patients' neutrophils show maturation arrest and excessive apoptosis, while T/B cells are reduced and functionally impaired. Patient-derived iPSCs, CRISPR-edited cells, and LCP1^+/L362F^ mice replicate these defects. Mechanistically, mutant LCP1 hyper-bundles F-actin, inducing VDAC1 oligomerization and mitochondrial apoptosis. This study further establishes LCP1 as a regulator of immune cell fate and suggests targeting actin dynamics as therapy.

## Introduction

The actin cytoskeleton plays a pivotal role in immune regulation by mediating cell migration, intercellular communication via surface receptors and soluble mediators, antigen processing, and nuclear functions.^1^, ^2^, ^3^, ^4^ Genetic mutations affecting actin cytoskeleton components lead to inborn errors of immunity. Actin-related inborn errors of immunity are collectively termed immuno-actinopathies.^5^^,^^6^ Currently, over twenty distinct immuno-actinopathy variants have been identified.^7^, ^8^, ^9^

Actin filaments (filamentous actin/F-actin) polymerize from globular actin monomers (globular actin/G-actin) in a polarized manner.^10^ Their remodeling is regulated by tissue-specific actin-binding proteins that mediate cellular functions.^11^ Lymphocyte cytosolic protein 1 (LCP1, also termed LPL, L-plastin, or plastin-2) mediates F-actin bundling.^12^ As a member of the plastin family, LCP1 exists alongside T-plastin and I-plastin isoforms, each demonstrating distinct tissue distribution.^13^ Notably, LCP1 is exclusively expressed in immune cells.^13^ Phosphorylation of LCP1 at Ser 5 promotes immune synapse formation during T lymphocyte activation.^14^^,^^15^ While LCP1 deficiency in mice preserves neutrophil development and migration, it impairs bacterial killing and integrin-mediated respiratory bursts.^16^ The clinical significance of LCP1 mutations remains under investigation. Mahat et al reported a heterozygous LCP1 mutation (c.694 A>T, p.I232F) associated with severe congenital neutropenia.^17^ Although *in vitro* studies characterized this mutation, the functional consequences of LCP1-mediated F-actin dysregulation on human neutrophil and lymphocyte development require further elucidation.

We report three patients from two unrelated families with heterozygous LCP1 missense mutations presenting with congenital neutropenia, combined immunodeficiency, and allergic manifestations. Laboratory analysis revealed increased apoptosis, cell cycle abnormalities, impaired migration, and defective differentiation/maturation in neutrophils and lymphocytes, while lymphocyte proliferation remained intact. Elevated cellular F-actin levels suggest that these LCP1 mutations represent gain-of-function variants. Thus, LCP1 mutations should be considered in congenital neutropenia and combined immunodeficiency cases.

## Materials and methods

### Study patients

All participants or their parents in this study provided written informed consent to participate in a protocol approved by the Ethical Review Board of the Children's Hospital of Chongqing Medical University, and all sample collection was performed in accordance with the Declaration of Helsinki.

### Whole-exome sequencing

For whole-exome sequencing, the TrueSeq Rapid exome kit and the Illumina HiSeq 3000 system were employed for analysis, as previously outlined.^18^ The data was sent to the Beijing MyGenostics Sequencing Company for analysis, using H19 as the reference genome.

### Sanger sequencing

Genomic DNA from the patients, parents, and siblings was used to validate and segregate the variants by Sanger sequencing. The following primers were designed for LCP1 amplification and sequencing: Forward: 5′-GACTTTCTGATTTTGGTGCAGGT-3'; Reverse: 5′-ATGAACGCTTTGTTAATGGACG-3'. The following primers were used for LCP1^+/L362F^ mouse sequencing identification: Forward: 5′-TTTCTCATGTTATGCCTGCTCACTC-3'; Reverse: 5′-CTTCGGGCCTGATTTTGGAATTG-3'. PCR products were sequenced by Sangon Biotech (Shanghai, China).

### Lymphocyte proliferation

A total of 1 × 10^6^ peripheral blood mononuclear cells (PBMCs) were labeled with carboxyfluorescein diacetate succinimidyl ester (CFSE) for cell dye and cell proliferation assessment.^19^ Phytohemagglutinin (PHA) or CD3/28 was employed to stimulate T lymphocytes, while F(ab)2′ IgM with CpG was used to stimulate B lymphocytes. The cells were incubated at 37 °C, 5% CO_2_, and 95% humidity for 72 h. The proliferation of T and B lymphocytes was evaluated using flow cytometry.

### Cell cycle detection

The cell cycle detection kit (Beyotime, C1052) was used for cell cycle detection. In brief, 1 × 10^6^ neutrophils or 2 × 10^5^ B cells were initially pre-cooled with 1 mL of ice bath and subsequently fixed with 70% ethanol at 4 °C for 2 h. Following fixation, cells were stained with propidium iodide in the dark at 37 °C for 30 min and treated with RNase A. Data acquisition was carried out using the Celesta instrument (BD Biosciences), and subsequent analysis was performed using FlowJo version 10.3.

### Apoptosis detection

To ascertain neutrophil apoptosis, 2 × 10^5^ cells were stimulated for 16 h at 37 °C in a 5% CO_2_ incubator in the presence or absence of 10 μg/mL of Fas-associated death domain (FAS). Apoptosis was detected using 7-AAD and Annexin V-FITC, or activated Caspase-3-FITC. For lymphocyte apoptosis, 5 μg/mL of CD3 and CD28 were employed to stimulate T cells, while 10 μg/mL of IgM was used to stimulate B cells. Cells were stimulated at 37 °C for 72 h. Before apoptosis detection, cells were initially stained with CD4, CD8, and CD19 for gating purposes.

### Migration assays

After HeLa cells had reached confluence in 6-well culture plates, uniform wound scratch lines were created within the designated locations using a pipette tip. Images of the scratches were captured at 0, 12, 24, and 48 h using an Upright fluorescence microscope (Nikon). The wound gaps were measured using ImageJ software (National Institutes of Health). For Transwell assays, 5 × 10^5^ PBMCs were seeded onto a 5 μm Transwell chamber (Corning) in the presence of scalar dilutions of stromal cell-derived factor 1 alpha (SDF1-α, Peprotech), and migration capacity was assessed after 4–6 h.^20^ For mouse bone marrow neutrophils, 5 × 105 cells were seeded onto a 5 μm Transwell chamber in the presence of scalar dilutions of CXC motif chemokine ligand 1 (CXCL1, Peprotech) or N-formyl-methionyl-leucyl-phenylalanine (fMLF, Sigma), and migration capacity was assessed after 4–6 h.

### Western blotting experiment

The cycloheximide chase assay was adapted from a previous report. Briefly, HEK293T cells were plated and transfected with wild-type (WT), L362F, and A365D plasmids. Twenty-four hours later, cycloheximide was added (100 ng/mL), and cells were collected at different time points for Western blotting detection. For G-Actin/F-Actin *in vivo* assay, fractionation of F and G actin was performed following the manufacturer's protocols (BK037 Cytoskeleton). For the analysis of the T cell receptor (TCR) signaling pathway, 1 × 10^6^ PBMCs were treated with or without 5 μg/mL CD3. PBMCs were lysed with protein splitting mixture, and then the supernatant was discarded by high-speed centrifugation to obtain protein. Proteins were separated by concentrated gel and separation gel, with LCP1 rabbit monoclonal antibody labeled, and an infrared imager was used for detection and analysis. Extracted cells were washed with phosphate-buffered saline solution and immediately lysed for 30 min in RIPA buffer containing the protease inhibitor, phenylmethylsulfonyl fluoride (PMSF, Sigma–Aldrich). Supernatants were centrifuged at high speed into protein loading buffer and denatured at 95 °C. Protein lysates were subjected to SDS-PAGE and transferred to PVDF membranes (Millipore, Germany) for analysis. For the detection of voltage-dependent anion channel 1 (VDAC1) oligomerization in mouse bone marrow neutrophils, Western blotting was performed using a primary antibody against VDAC1. In experiments involving inhibitors, they were administered 1 h (DIDS, Latrunculin B) and 12 h (Oroxylin A) before cell collection.

### Confocal microscope

CD4^+^ T cells were isolated from PBMCs using a separation kit (StemCell Technologies™) following standard protocols. The selected cells were suspended in a phosphate-buffered saline solution containing 2% fetal bovine serum and placed onto poly-l-lysine-coated slides for 45 min. CD4^+^ T cells were stimulated with mouse anti-human CD3 (Biolegend) and mouse anti-human CD28 (Biolegend) at 37 °C. The stimulation was halted at 0, 5, 10, and 20 min by adding 4% paraformaldehyde. Rabbit anti-human LCP1 primary antibodies were incubated overnight, F-actin was stained with Phalloidin-iFluor 488 (1:1000) (Abcam), and the cell nuclei were stained with DAPI (Beyotime). For neutrophils, cells were stimulated in the presence of 100 nM fMLF for 0, 1, 3, and 5 min. Analysis was carried out using a Nikon A1 confocal microscope. The average fluorescence intensity of each protein was measured using NIS-Elements AR 3.2 software. For immunofluorescence staining of mouse bone marrow neutrophils or induced pluripotent stem cell (iPSC)-derived neutrophils, cells were stained with primary antibodies against translocase of outer mitochondrial membrane 20 (Tomm20) and VDAC1 for mitochondrial visualization and phalloidin for F-actin labeling; for inhibitor-treated groups, specific inhibitors were administered 1 h (DIDS, Latrunculin B) and 12 h (Oroxylin A) before cell collection, with all other analytical procedures performed as previously described.

### iPSCs and neutrophil differentiation

PBMCs were cultivated in StemSpan™ SFEM II medium (STEMCELL Technologies) supplemented with several cytokines (stem cell factor, FLT-3, IL-3, and IL-6) for four days. Subsequently, the reprogramming procedure was executed using the CytoTune™-iPS 2.0 Sendai Reprogramming Kit (ThermoFisher Scientific).^21^ The transduced cells were subsequently transferred to plates coated with Matrigel (Corning Incorporated) and cultured in phosphoglucomutase 1 (PGM1) medium (Cellapy). After approximately 20 days, colonies resembling iPSCs were selected and expanded using PGM1 medium. For the differentiation of iPSCs into neutrophils, iPSCs were initially induced into CD34^+^ hematopoietic progenitor cells using the STEMdiff Hematopoietic Kit (STEMCELL Technologies). Next, CD34^+^ cells were resuspended in StemSpan SFEM II with 100 ng/mL of human stem cell factor, human thrombopoietin, and human Fms-like tyrosine kinase 3-ligand (PeproTech). Granulocytic differentiation was assessed by culturing cells in StemSpan SFEM II medium with 3 ng/mL granulocyte-colony stimulating factor (G-CSF), 10 ng/mL of stem cell factor, and 10% fetal calf serum for 10–14 days.^22^^,^^23^ Granulocyte colony-forming unit assays were conducted using MethoCult H4230 (STEMCELL Technologies) supplemented with G-CSF and stem cell factor.^24^

### single-cell RNA-sequencing data analysis

Cryopreserved bone marrow mononuclear cells were isolated from patient 1 and two healthy controls (HCs) using Ficoll-Hypaque density gradient centrifugation. Single-cell RNA-sequencing libraries were prepared using 10 × Genomics Chromium Controller (v3 chemistry) following manufacturer protocols.

Raw sequencing data were processed with Cell Ranger (v6.1.2), with reads aligned to GRCh38 to generate feature-count matrices. Samples were merged initially to minimize batch effects. Cells containing ≥ 100 detected features were retained. Using Seurat (v4.3.0.1), we performed principal components analysis on the top 3000 variable features, followed by dimensional reduction and clustering (resolution = 2) using the top 30 principal components. After excluding clusters lacking cell-type-specific markers, 39,028 cells remained for analysis. Notably, HC2 was a 2.8-year-old male undergoing bone marrow collection during hospitalization for juvenile idiopathic arthritis, without evidence of immunodeficiency. Differential expression analysis employed Wilcoxon Rank Sum tests with Bonferroni correction. Significant genes underwent functional enrichment analysis via Metascape. Cell cycle phase assignment was based on module scores for S and G2M phase genes. G1 phase cells were identified from scatter plots of G2M versus S scores, with remaining cells classified as S or G2M phase based on established thresholds.

### LCP1^+/L362F^ mice

C57BL/6 *LCP1*^+/L362F^ knock-in mice were generated using CRISPR/Cas9-mediated genome engineering by Cyagen Bioscience. In brief, the gRNA to the mouse Lcp1 gene, the donor vector containing "KI region-p.L362F(TTG to TTC)" cassette, and Cas9 mRNA were co-injected into fertilized mouse eggs to generate targeted knock-in offspring. F0 founder animals were identified by PCR followed by sequence analysis, which were bred to WT mice to test germline transmission and F1 animal generation.

gRNA target sequences were as follows: gRNA1 (matching forward strand of gene): GTTTACCACTGGCAGTATGGAGG; gRNA2 (matching reverse strand of gene): ATCAACCTCCATACTGCCAGTGG; gRNA3 (matching reverse strand of gene): GGGTGCATGCCGTGTGTGGCTGG; gRNA4 (matching reverse strand of gene): GACTGGGTGCATGCCGTGTGTGG.

All animal work was reviewed and approved by the Institutional Animal Care and Usage Committee of Children's Hospital of Chongqing Medical University.

### Statistical analysis

Data analysis was performed using GraphPad Prism 8.0 (GraphPad Software, USA), except for flow cytometry data, which were analyzed using FlowJo 10.3 (TreeStar Inc, USA). Statistical tests used were described in the figure legends. Statistical significance was defined as *P* < 0.05.

## Results

### Clinical manifestation

#### *Family A*

**Figure 1:**
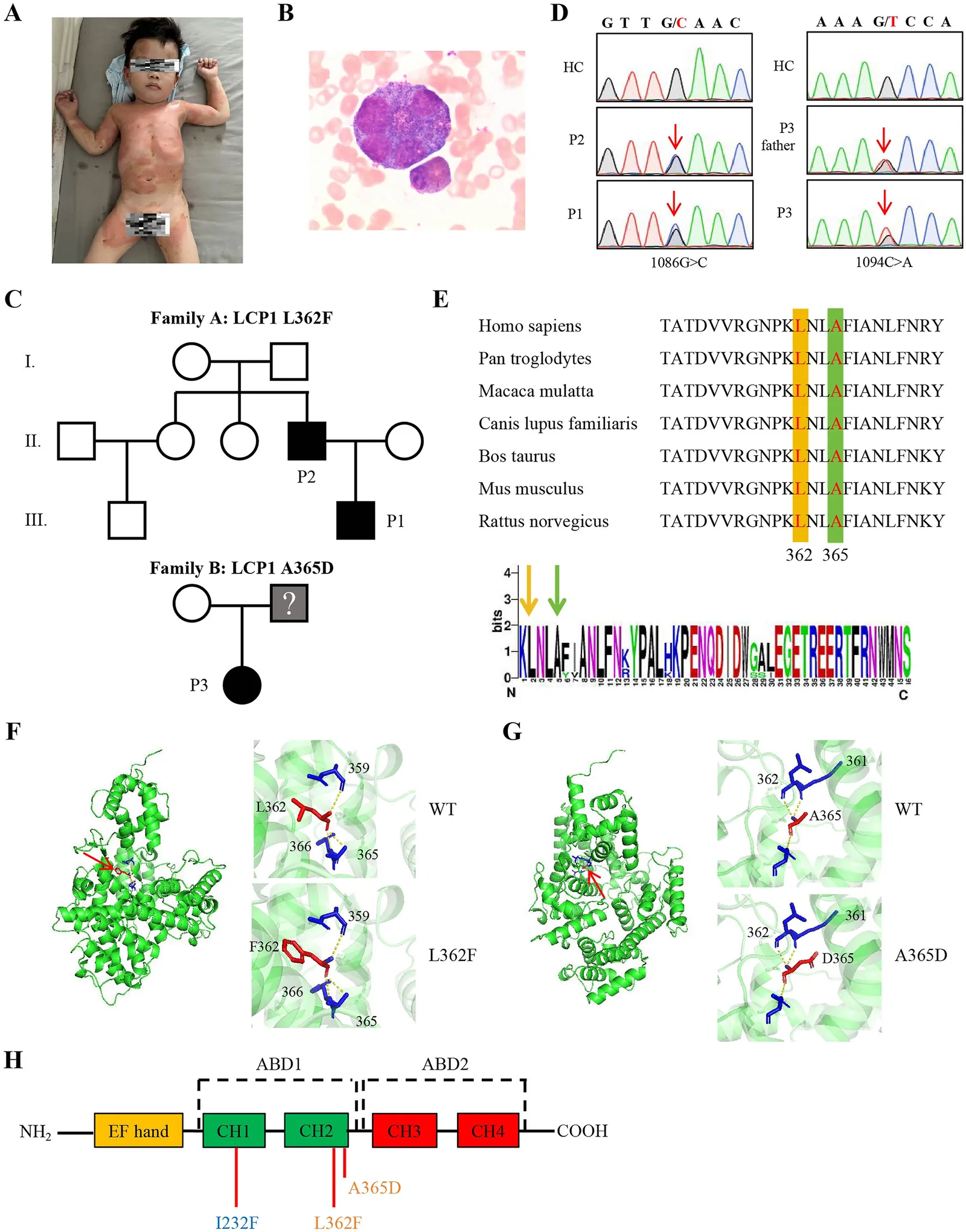
Identification of heterozygous pathogenic mutations in the LCP1 gene in three patients with neutropenia and lymphopenia. **(A)** Systemic allergic urticaria in P1 at age 2. **(B)** Bone marrow aspirate smear of P3 demonstrating granulocytic maturation arrest at promyelocyte/myelocyte stages and giant tetraploid karyotype neutrophils (Wright's stain, × 1000). **(C)** Pedigrees of families A and B: affected individuals are shown in black; mutation carriers with uncertain phenotypes are shown in grey with question marks. **(D)** Sanger sequencing of LCP1 mutation sites in families A and B, with mutated bases indicated by red font or arrows. P1 and P2 carry LCP1 c.1086G>C mutations. P3 and her father carry LCP1 c.1094C>A mutations. **(E)** Amino acid residues conservation analysis of LCP1 mutations: L362 (yellow) and A365 (green). These two amino acid sites are highly conserved across different species. **(F, G)** AlphaFold2-predicted 3D structure of LCP1 showing L362F and A365D within the horseshoe structure without disrupting hydrogen bonds, but increasing structural stability with potential actin-binding effects. **(H)** Schematic of LCP1 protein domains with patient variants (red underline, yellow font) and previously reported I232F (blue font).

The index case (P1) is a 4-year-old male born to non-consanguineous parents. Samples were collected at age 2 years (born in June 2021). Growth and development were normal. Medical history included severe methicillin-resistant *Staphylococcus aureus* pneumonia requiring neonatal intensive care unit admission during the neonatal period, and hospitalization for rotavirus enteritis at 8 months. Five pneumonia episodes (respiratory syncytial virus, human rhinovirus, influenza virus, *Streptococcus pneumoniae*, and methicillin-resistant *Staphylococcus aureus*) required hospitalization but not intensive care (Table 1). Recurrent oral aphthous ulcers were noted. Hematologic evaluation revealed leukocytopenia, neutropenia, and lymphocytopenia, without anemia or thrombocytopenia. Absolute neutrophil count remained persistently below 0.5 × 10^3^/uL despite lymphocyte count fluctuations (Table S1). Serum IgA and IgM levels were decreased (Table 2). Initial evaluation for suspected severe congenital neutropenia showed no evidence of cyclicity on twice-weekly complete blood counts. Anti-neutrophil antibody testing was negative. Bone marrow examination at 3 months demonstrated hypocellularity with dysgranulopoiesis (myeloid-to-erythroid ratio: 1.3:1), showing granulocytic maturation arrest at promyelocyte and myelocyte stages (Fig. S1A; Table 1). G-CSF therapy (5 μg/kg) increased neutrophil count above 1.0 × 10^3^/uL, indicating a favorable response to G-CSF. Combined G-CSF and regular intravenous immunoglobulin therapy reduced infection frequency and severity. Repeat bone marrow examination at age 2.5 years showed a normal myeloid/erythroid ratio with a left-shifted granulocytic series and maturation up to band and segmented neutrophils, indicating resolution of prior granulocytic hypoplasia. Additional findings included severe allergic urticaria requiring oral steroids (Fig. 1A), incomplete left testicular descent, and right hydrocele. Vaccinations (hepatitis B, DTP, poliomyelitis, influenza) were tolerated with occasional mild fever. No live vaccines were administered.

**Table 1:** Clinical features of patients with LCP1 mutations.

	P1	P2	P3
Age at first symptoms	18 days	6 years	7 months
Viral infections	Recurrent respiratory tract infections, respiratory syncytial virus, human rhinovirus, and influenza virus pneumonia. Rotavirus, COVID-19 infection	Recurrent pneumonia, hepatitis B virus and COVID-19 infection, cutaneous (severe VZV); gastrointestinal (hepatitis B)	Influenza virus pneumonia, COVID-19 infection, recurrent respiratory tract infections
Bacterial infections	Methicillin-resistant *Staphylococcus aureus*, *Streptococcus pneumoniae* pneumonia	*Mycobacterium tuberculosis*, *Haemophilus influenzae* pneumonia, otitis media	*Staphylococcus aureus*, *Streptococcus pneumoniae* pneumonia, gluteal cellulitis
Hematology	Leukopenia, lymphopenia, and neutropenia	Leukopenia, lymphopenia, and neutropenia, splenomegaly	Leukopenia, lymphopenia, and neutropenia, moderate anemia, swollen lymph nodes
Neurology		Sensory sensorineural hearing loss	Wide extracerebral space
Skin and mucous membranes	Aphthous ulcers	Recurrent oral ulcers, granulomatous skin disease, gingivitis, delayed wound healing	Aphthous ulcers
Urology	Left incomplete testicular descent, right hydrocele		
Allergy	Eczema, urticaria, allergic rhinitis, epistaxis	Eczema, urticaria	Eczema
Bone marrow cytology	Hypocellularity with dysgranulopoiesis, granulocytic maturation arrest at the promyelocyte and myelocyte stages	Decreased segmented neutrophils, along with increased proportions of promyelocytes and myelocytes, impaired granulocytic maturation	Granulocytic maturation arrest at promyelocyte/myelocyte stages, with 12% multinucleated immature granulocytes. The size and shape of the young erythrocytes were normal. Some mature red blood cells are small and full of pigment.

**Table 2:** Laboratory data for patients with LCP1 mutation at the time of genetic diagnosis.

	P1	P3	Reference	P2	Reference
Age at test	1y2m	9m		34y	
White blood cell count	1.43 (L)	1.52 (L)	5.5–13.6	1.65 (L)	4–10
Hemoglobin (g/dL)	106	76 (L)	104–143	96 (L)	120–160
Platelet count ( × 10^3^/μL)	170	366	100–516	205	100–300
Absolute lymphocyte count ( × 10^3^/μL)	0.93 (L)	1.27 (L)	2.70–9.10	0.69 (L)	0.8–4
Absolute neutrophil count ( × 10^3^/μL)	0.35 (L)	0.05 (L)	0.90–5.50	0.78 (L)	2–7.7
CD3^+^ cells (%)	81.7 (H)	78.9 (H)	53.88–72.87	83.3 (H)	66.9–83.1
CD3^+^CD8^+^ cells (%)	19.8	31.13	19.00–32.51	32.30	20.4–34.7
CD3^+^CD4^+^ cells (%)	53.9 (H)	44.2 (H)	24.08–42.52	39.30	33.19–47.85
CD19^+^ cells (%)	7.7 (L)	11.5 (L)	13.23–26.39	ND	
CD3^–^CD56^+^ cells (%)	10.3	8.09	7.21–20.90	ND	
CD4/CD8	2.7 (H)	1.42	0.90–2.13	1.22	0.97–2.31
CD3^+^ cells per μL	760.1 (L)	ND	1400–8000	ND	
CD3^+^CD8^+^ cells per μL	183.9 (L)	ND	580–1735	43.6 (H)	21.96–36.80
CD3^+^CD8^+^CD27^+^CD45RA^+^ cells per μL	150.2 (L)	ND	356–1095	10.3 (L)	35.34–72.32
CD3^+^CD8^+^CD27^+^CD45RA^–^ cells per μL	17.1 (L)	ND	56–406	23.8	10.96–31.00
CD3^+^CD8^+^CD27^–^CD45RA^–^ cells per μL	0.3 (L)	ND	6–145	9.9	2.38–15.8
CD3^+^CD8^+^CD27^–^CD45RA^+^ cells per μL	16.3	ND	9–406	56.0 (H)	5.08–31.24
CD3^+^CD4^+^ cells per μL	500.9 (L)	ND	902–2253	38.6	22.25–39.00
CD3^+^CD4^+^CD27^+^CD45RA^+^ cells per μL	390.7 (L)	ND	472–1760	9.5 (L)	39.5–66.26
CD3^+^CD4^+^CD27^+^CD45RA^–^ cells per μL	98.2 (L)	ND	212–735	66.1 (H)	25.34–49.90
CD3^+^CD4^+^CD27^–^CD45RA^–^ cells per μL	5.0 (L)	ND	15–87	24.0 (H)	4.68–15.70
CD3^+^CD4^+^CD27^–^CD45RA^+^ cells per μL	6.7	ND	0–22	0.5	0.00–1.54
CD19^+^ cells per μL	71.5 (L)	ND	461–1456	ND	
CD19^+^IgD^+^CD27^–^ cells per μL	54.4 (L)	ND	323–1089	61.3	53.78–78.64
CD19^+^IgD^–^CD27^+^ cells per μL	8.0 (L)	ND	26–124	25.4 (H)	7.15–23.10
CD19^+^CD38^+^CD24^+^ cells per μL	12.5 (L)	ND	35–127	12.7 (H)	1.38–9.42
CD19^+^CD38^+^CD24^–^ cells per μL	4.6 (L)	ND	4–63	8.2 (H)	0.49–7.06
CD3^–^CD56^+^ cells per μL	95.4 (L)	ND	270–1053	ND	
Serum IgG (mg/dL)	5.53	6.73	3.41–19.6	9.83	8–15.5
Serum IgA (mg/dL)	0.1 (L)	0.30	0.19–2.20	1.7	0.83–2.9
Serum IgM (mg/dL)	0.1 (L)	0.49	0.43–1.63	0.45 (L)	0.7–2.2
Serum IgE (mg/dL)	243 (H)	ND	<60	574 (H)	0.1–150
T-cell receptor excision rings (copies/μL blood)	24.9	ND	>40	ND	
Kappa-deleting excision rings (copies/μL blood)	9.2	ND	>20	ND	

Note: For each lymphocyte subgroup of P2, only the proportion of each cell population was detected.

P2 (father of P1) had *Haemophilus influenzae* pneumonia, otitis media, recurrent oral ulcers, gingivitis, delayed wound healing, and allergies. Pulmonary tuberculosis was diagnosed at age 23. Whole-exome sequencing revealed no pathogenic mutations in Mendelian susceptibility to mycobacterial diseases (MSMD)-associated genes. Leukocyte, lymphocyte, and neutrophil counts were decreased. No other hematological abnormalities were observed. Bone marrow aspiration at age 22 showed decreased segmented neutrophils with increased promyelocytes and myelocytes, suggesting impaired granulocytic maturation (Table 1). Serial neutrophil measurements excluded cyclic neutropenia, and anti-neutrophil antibody tests were negative. Persistent neutrophil counts fluctuate within 0.5–1.0 × 10^3^/uL, indicating chronic moderate neutropenia (Table S1). Without regular G-CSF therapy, he developed mild bronchiectasis and recurrent oral ulcers.

#### *Family B*

P3, a 4-year-old female, is the first child of non-consanguineous parents with normal growth parameters. At 7 months, she developed severe *Staphylococcus aureus* pneumonia requiring one month of hospitalization for intravenous antibiotics. Laboratory tests showed iron deficiency anemia (hemoglobin 76 g/L), neutropenia, and lymphocytopenia, without thrombocytopenia (Table S1). Bone marrow cytology at 9 months revealed granulocytic maturation arrest at promyelocyte/myelocyte stages, with 12% multinucleated immature granulocytes (Fig. 1B; Table 1). Repeat bone marrow examination at age 4 during gluteal cellulitis demonstrated hypoplastic granulopoiesis (myeloid-to-erythroid ratio: 0.29:1) with dysplasia and maturation arrest (Fig. S1B). The patient developed recurrent oral ulcers without regular G-CSF therapy. Clinical courses included three severe pneumonias (*Staphylococcus aureus*, *Streptococcus pneumoniae*, influenza virus), otitis media, and frequent upper respiratory infections (> 5/year) requiring intravenous antibiotics.

### Identification of pathogenic LCP1 mutations

Whole-exome sequencing of P1 and family members revealed no severe congenital neutropenia-associated mutations per the 2024 update of the phenotypic classification by the International Union of Immunological Societies (IUIS) expert committee on inborn errors of immunity.^25^ A novel heterozygous LCP1 c.1086G>C (p.L362F) mutation was identified in P1 and P2, co-segregating with the disease phenotype (Fig. 1C and D). P3 carried a distinct heterozygous LCP1 c.1094C>A (p.A365D) mutation. Both variants exhibited high CADD pathogenicity scores (25.6 and 32, respectively) and were extremely rare (allele frequency < 0.0001) in the 1000 Genomes Database. Multiple prediction tools, including AlphaMissense, classified these mutations as deleterious/pathogenic (Table S2).^26^ The affected residues were evolutionarily conserved (Fig. 1E). AlphaFold2 analysis indicated preserved hydrogen bonding without structural disruption (Fig. 1F and G). Both mutations localized to a horseshoe-shaped F-actin interaction domain, adjacent to the previously reported I232F mutation within LCP1's actin-binding domain 1 (ABD1), though in distinct CH domains (Fig. 1H).^17^ Collectively, these data provide strong evidence implicating these LCP1 mutations in disease pathogenesis.

### Impaired neutrophil differentiation, increased apoptosis, and cell cycle arrest

**Figure 2:**
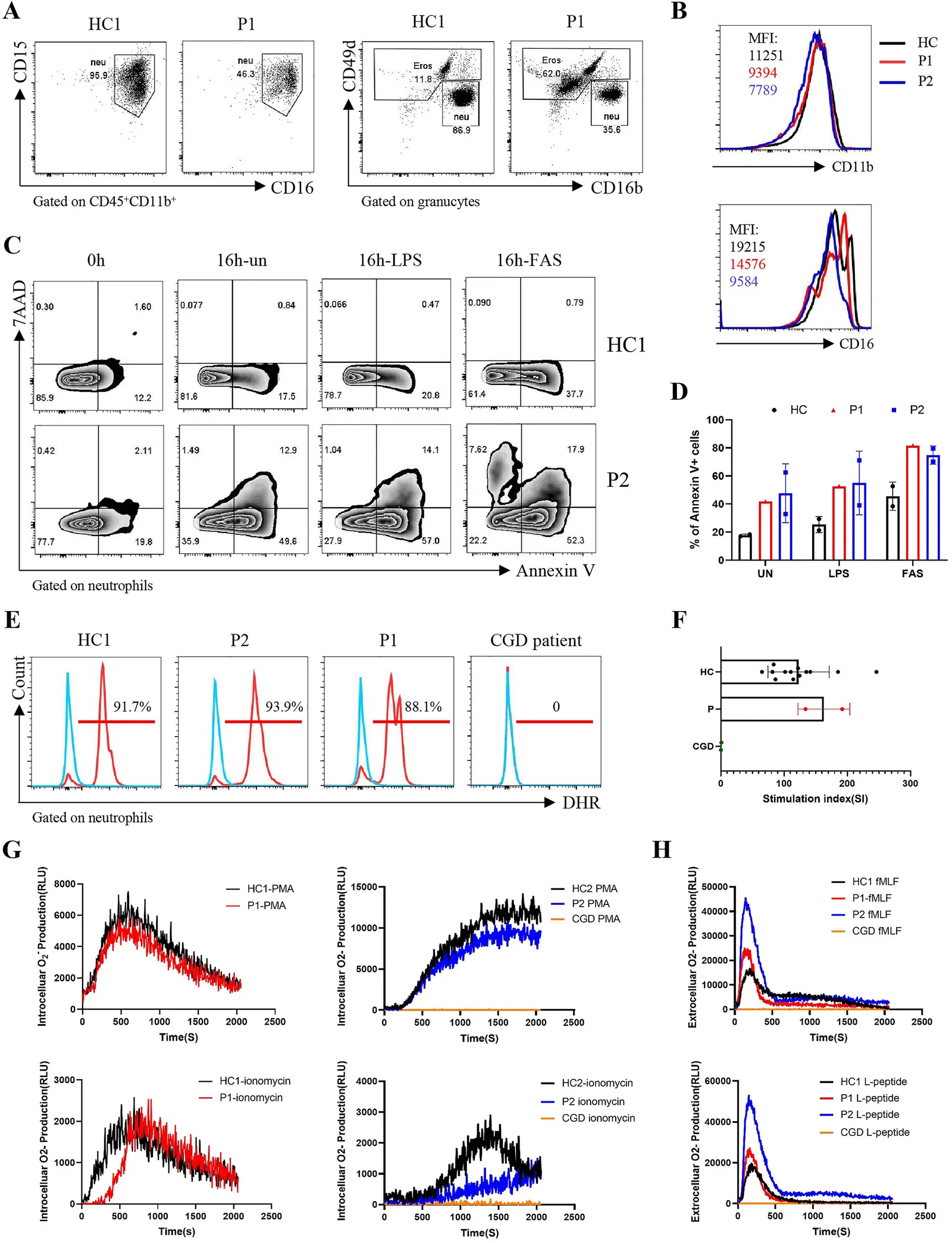
Patient peripheral blood neutrophils exhibit elevated apoptosis, dysregulated reactive oxygen species (ROS) production, and reduced surface maturation markers. **(A)** Flow cytometry revealed decreased neutrophil (CD15^+^CD16^+^ or CD16^+^CD49d^low^) proportion and increased eosinophil (CD16b^low^CD49d^+^) proportion in P1. **(B)** Mean fluorescence intensity (MFI) of neutrophil surface maturation markers CD16 and CD11b was reduced in P1 (red) and P2 (blue). **(C, D)** In P1 and P2 LPS- or FAS-stimulated neutrophils, early (Annexin V^+^7-AAD^–^) and late (Annexin V^+^7-AAD^+^) apoptosis were elevated after 16 h incubation. The bar graph illustrates the proportion of Annexin V-positive cells in the UN (unstimulated) group and the FAS or LPS stimulation group. **(E, F)** Dihydrorhodamine (DHR) staining demonstrated normal respiratory burst levels in patient neutrophils, with the CGD patient as a negative control. The statistical plot indicates that the patients exhibit a stimulation index (stimulated group MFI/unstimulated group MFI) comparable to that of the healthy controls (HCs). **(G, H)** Quantification of endogenous and exogenous ROS levels in P1 (red) and P2 (blue) neutrophils. The data represent one of two independent experiments.

Given that neutropenia was the predominant feature in all patients, we performed comprehensive neutrophil analyses. Peripheral blood smears from P1 and P2 revealed elevated immature band neutrophils, along with increased eosinophil proportions. Flow cytometry demonstrated reduced mature neutrophil (CD16b^+^CD49d^–^) percentages and decreased CD11b/CD16 surface expression compared with HCs (Fig. 2A and B), consistent with our previous observations in patients with severe congenital neutropenia 1.^27^ Functional assessments showed significantly higher apoptosis rates in patient neutrophils following 16 h *in vitro* stimulation with/without FAS or lipopolysaccharide (LPS) versus HCs (Fig. 2C and D). While dihydrorhodamine (DHR) staining revealed comparable respiratory burst capacity (Fig. 2E and F), enzyme assays indicated altered reactive oxygen species (ROS) metabolism: diminished intracellular O_2_^–^ production but elevated extracellular O_2_^–^ generation, suggesting dual dysregulation of endogenous and exogenous ROS pathways (Fig. 2G and H).

**Figure 3:**
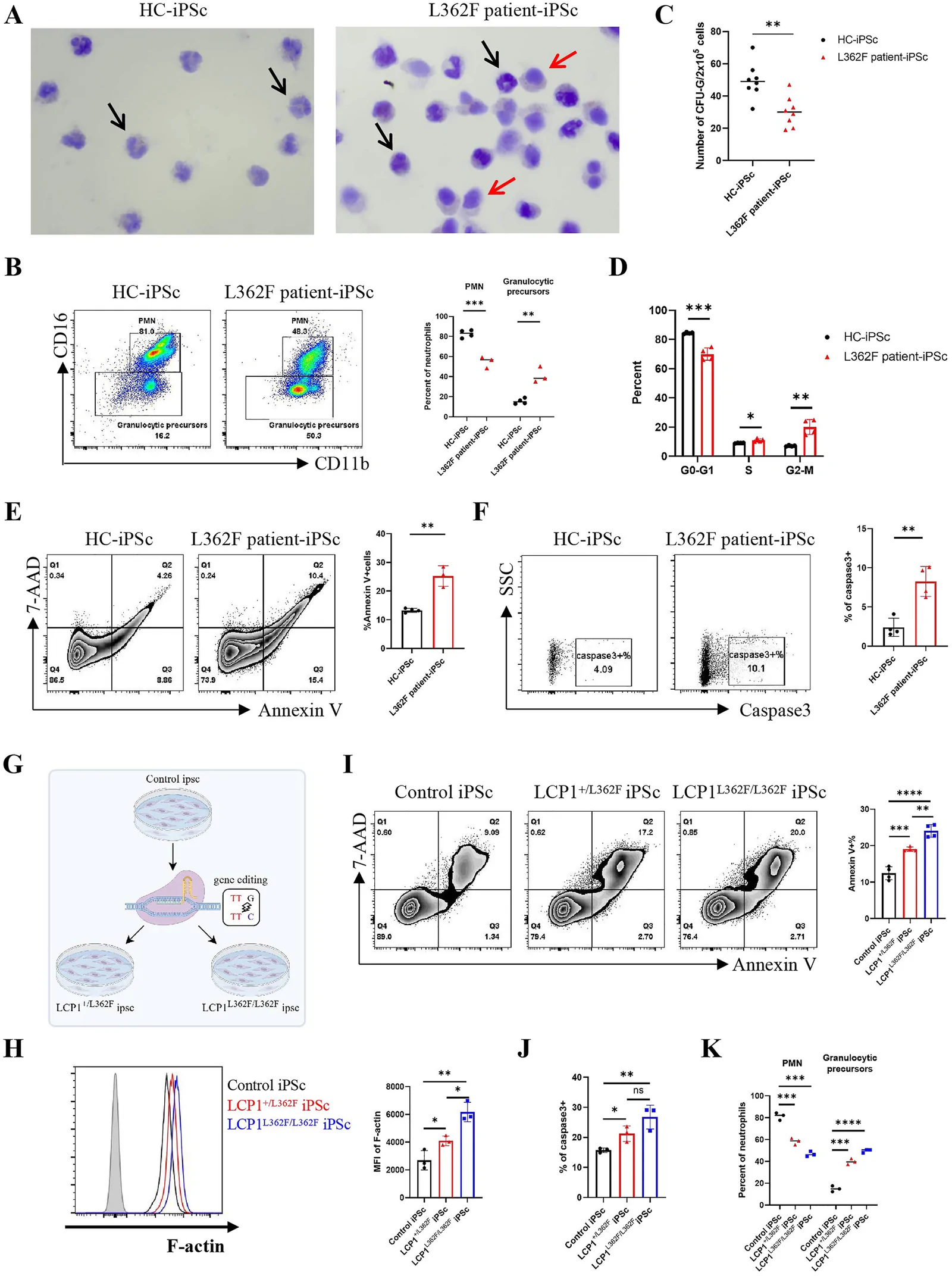
Induced pluripotent stem cells (iPSCs) from the L362F patient (P1) and gene-edited iPSCs from a healthy control demonstrated impaired neutrophil differentiation, cell cycle arrest, and increased apoptosis. **(A)** Wright-Giemsa staining of cytospin preparations at day 12–14 (100 × magnification). The black arrows indicate mature, multinucleated neutrophils, while the red arrows indicate immature juvenile neutrophils. **(B)** Flow cytometry analysis of CD11b^+^CD16^+^ polymorphonuclear neutrophils (PMN) and CD11b^−/+^CD16^–^ granulocyte precursors, with quantification in the right panel. Data represent 3 or 4 independent experiments (mean ± standard error of the mean/SEM). Two-way ANOVA with Sidak multiple comparison test was used to determine significance. **(C)** Granulocyte colony-forming unit (CFU-G) count per 20000 CD34^+^ cells after HSPC culture for 5 days. **(D)** Flow cytometry analysis of the cell cycle during *in vitro* differentiation of CD34^+^ hematopoietic stem cells into neutrophils at day 7. **(E, F)** Percentage of Annexin V^+^ or caspase-3^+^ granulocyte progenitors at day 7. Data represent 3–5 independent experiments (mean ± SEM). *P*-values calculated by a two-sided unpaired *t*-test. **(G)** CRISPR-Cas9 strategy for generating isogenic iPSC lines with homozygous/heterozygous LCP1 L362F mutations. **(H)** F-actin expression on LCP1^+/L362F^ (red) and LCP1^L362F/L362F^ (blue) iPSC-derived neutrophils (day 12–14). **(I, J)** Annexin V^+^ and caspase-3^+^ granulocyte progenitors on day 7. Data represent 3 independent experiments (mean ± SEM). **(K)** Identification of PMN and granulocyte precursors in LCP1^+/L362F^ and LCP1^L362F/L362F^ iPSC-derived neutrophils (day 12–14). Data represent 3 or 4 independent experiments (mean ± SEM). Two-way ANOVA with Sidak multiple comparison test was used to determine significance.

To further investigate granulocyte developmental and functional defects in patients, we established an *in vitro* disease model using P1-derived induced pluripotent stem cells (L362F patient-iPSCs). These iPSCs were differentiated into CD34^+^ hematopoietic progenitor cells following the protocol by Hiramoto et al (Fig. S2A–S2D).^23^ Following 10–14 days of G-CSF treatment, L362F patient-iPSCs yielded fewer mature neutrophils but more precursor cells compared with HC iPSCs (Fig. 3A and B). Myeloid differentiation was assessed using hematopoietic colony-forming assays.^24^ L362F patient-iPSCs demonstrated impaired myeloid differentiation, with significantly fewer granulocyte colony-forming unit colonies than HC iPSCs (Fig. 3C; Fig. S2E). Additionally, L362F patient-iPSCs-derived neutrophil precursors showed increased apoptosis and cell cycle abnormalities (Fig. 3D–F). These findings demonstrate neutrophil differentiation and maturation defects associated with elevated apoptosis and cell cycle arrest.

To exclude potential confounding effects of other unknown gene mutations, we introduced the patient-specific variant (LCP1 c.1086G>C) into control iPSCs, generating heterozygous LCP1^+/L362F^ and homozygous LCP1^L362F/L362F^ iPSCs (Fig. 3G; Fig. S2F). These isogenic cell lines reproduced key phenotypes observed in patient-derived iPSCs, including F-actin aggregation, elevated apoptosis, and mild neutrophil differentiation defects (Fig. 3H–K; Fig. S2G). Phenotypic severity correlated with mutation dosage, being more pronounced in homozygous lines.

### Lymphocyte subset abnormalities and functional defects

P1 exhibited decreased absolute counts of T, B, and NK cells (Table 2). P2 demonstrated a shift from naive to activated T and B cell populations, and he showed elevated expression of aging markers (CD57, HLA-DR, PD-1) on CD4^+^ and CD8^+^ T cells compared with HCs (Fig. S3A and S3B). CXCR5^+^CD45RO^+^ Tfh, CD25^+^CD127^–^ Tfr, and CD25^+^FOXP3^+^ Treg cells were reduced in P1 and P2 versus HCs (Fig. S3C–S3F), with Tfh subsets skewed toward Th2/Th2-like phenotype (Fig. S3G). P2 maintained normal NK cell CD107a production (Fig. S3H), suggesting combined immunodeficiency with B cell cytopenia and Tfh dysregulation.

**Figure 4:**
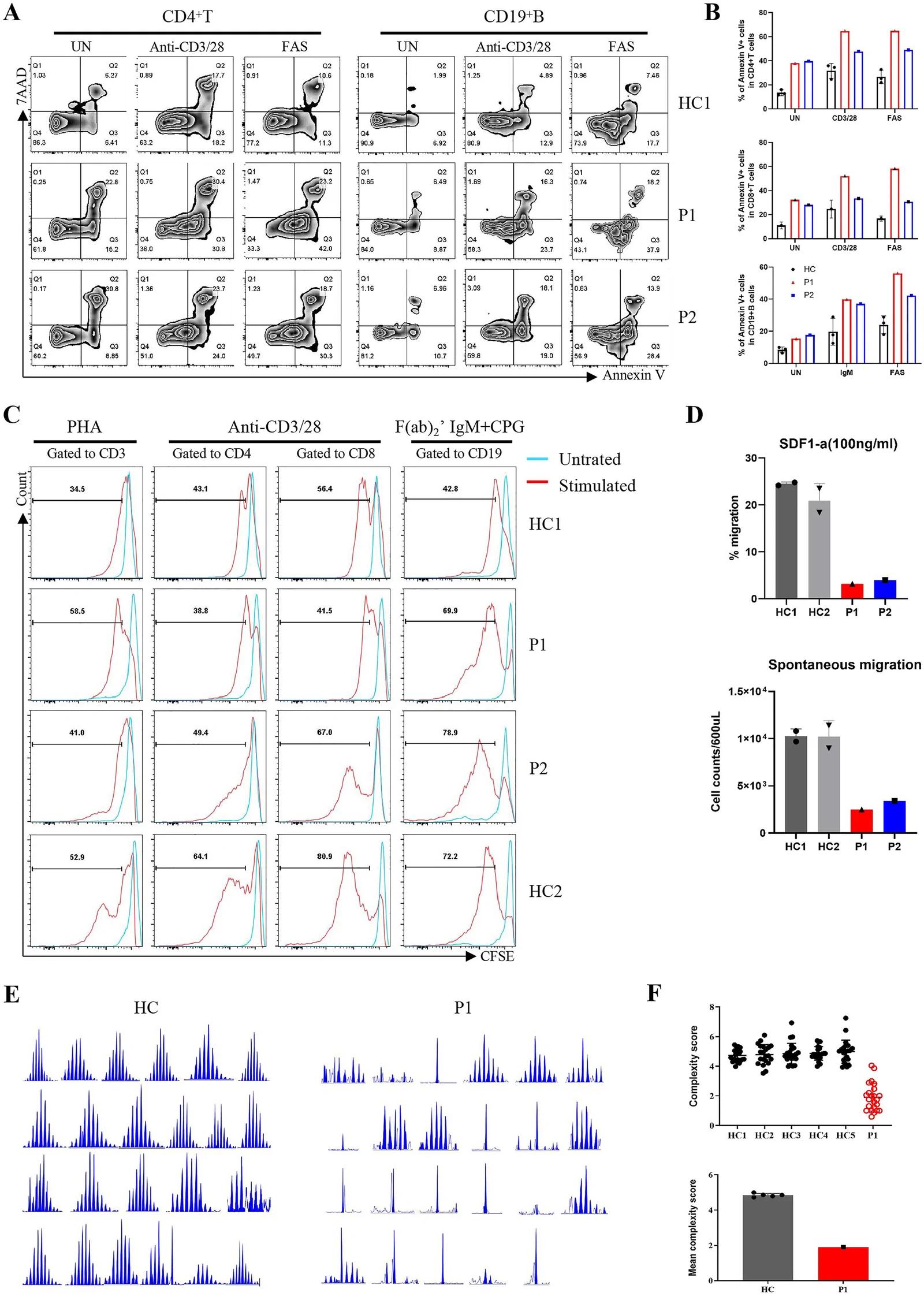
Immunological phenotypes of T and B lymphocytes in LCP1 mutation patients. **(A, B)** Increased apoptosis of CD4^+^ and CD8^+^ T lymphocytes under unstimulated conditions or following stimulation with anti-CD3/28 or FAS. Enhanced apoptosis of CD19^+^ B cells after stimulation with F(ab)_2_' IgM or FAS. **(C)** Proliferation capacity of T and B lymphocytes from P1 and P2 measured by CFSE staining. T cell proliferation was induced by PHA or anti-CD3/28, while B cell proliferation was stimulated with F(ab)_2_' IgM. **(D)** Spontaneous and SDF-1α-induced (100 ng/mL) migration of PBMCs assessed using Transwell assays with 5 μm membranes. **(E)** CDR3 size distribution in TCR-Vβ subfamilies of P1. Healthy controls displayed a Gaussian distribution across all 23 TCR-Vβ subfamilies, whereas the patient exhibited oligoclonal peaks. **(F)** Complexity scores comparing P1 with healthy controls.

Lymphocyte functional assessment revealed increased apoptosis in P1 and P2 CD4^+^ T cells following anti-CD3/CD28 or FAS stimulation, and in B cells after anti-IgM or FAS stimulation (Fig. 4A and B). In contrast, mitogen-induced proliferative responses remained comparable to HCs (Fig. 4C). PBMC migration was significantly impaired in both spontaneous and SDF1-α-induced conditions (Fig. 4D). TCR repertoire analysis showed monoclonal/oligoclonal peaks in P1 (Fig. 4E and F), with reduced T-cell receptor excision rings (TRECs) and kappa-deleting excision rings (KRECs) versus controls (Table 2). Intriguingly, P2 T cells exhibited normal up-regulation of activation markers (CD69, CD25, ICOS) and preserved cytokine production (IL-17A, IFN-γ, IL-4, IL-2) following stimulation (Fig. S3I–S3K). TCR signaling appeared intact in P1 (Fig. S3L). These findings indicate that lymphocytopenia may result from enhanced apoptosis and impaired lymphocyte homing, reflecting T cell subset homeostasis disruption.

### LCP1 L362F and A365D are gain-of-function mutations

**Figure 5:**
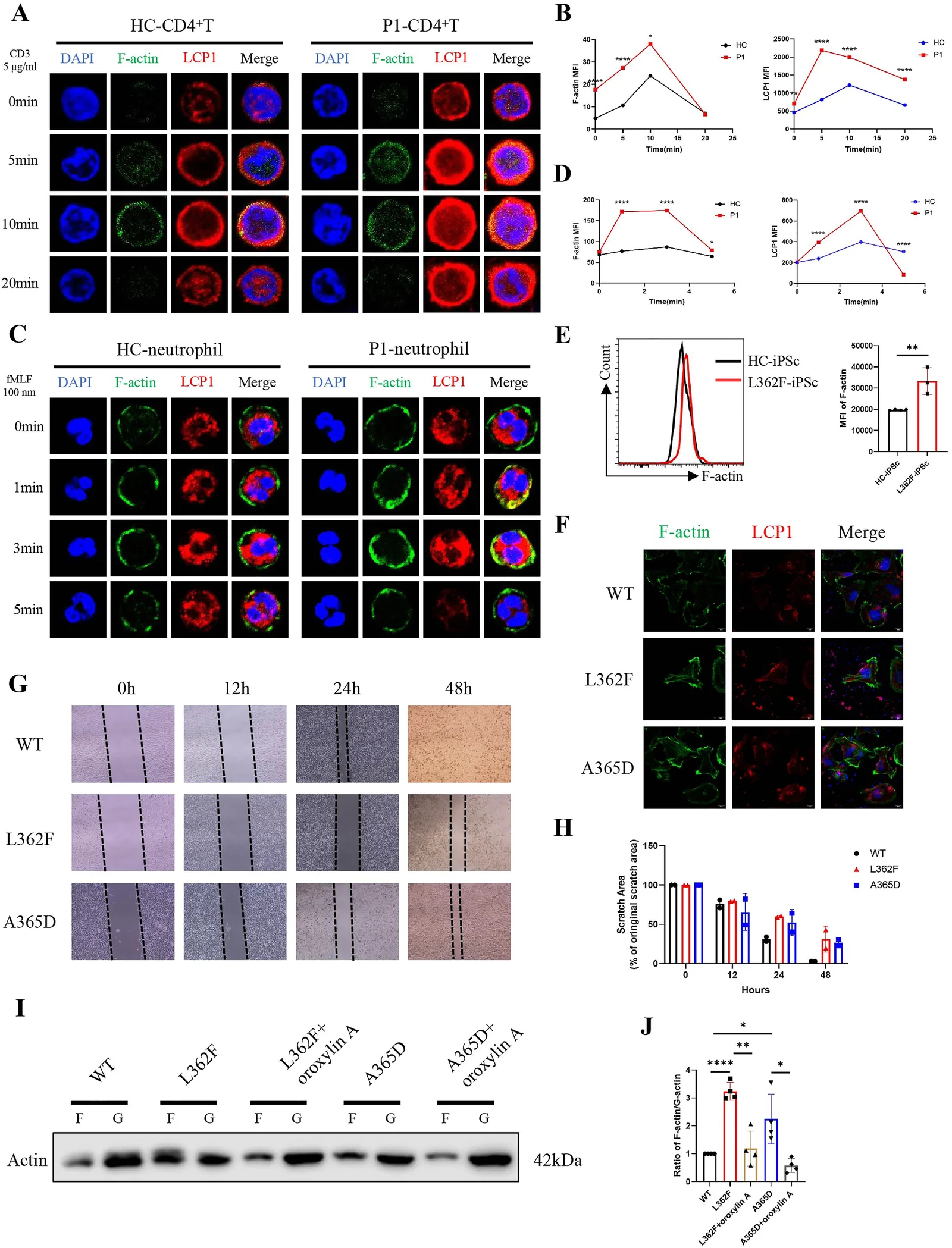
F-actin hyper-polymerization in primary lymphocytes, neutrophils, iPSC-derived neutrophils, and HeLa cells expressing LCP1 L362F and A365D. **(A, C)** Confocal microscopy images of F-actin and LCP1 expression in CD4^+^ T cells (anti-CD3 stimulated) from P1 (A) and neutrophils (fMLF stimulated) (C). **(B, D)** Mean fluorescence intensity (MFI) of F-actin and LCP1 quantified using NIS-Elements AR 3.2 software (*n* > 50 cells/group). **(E)** Flow cytometry analysis of F-actin expression in iPSC-derived mature neutrophils (day 12–14). Three independent replicate experiments. *P*-values were calculated using Student's *t*-test. **(F)** Confocal microscopy of HeLa cells transfected with WT, L362F, or A365D plasmids, stained with anti-LCP1 antibodies (red) and phalloidin (green) (scale bar: 10 μm). Blue, DAPI. **(G)** Scratch assay in HeLa cells transfected with LCP1 L362F and A365D plasmids. **(H)** Scratch area quantification by ImageJ at 0, 12, 24, and 48 h (*n* = 2 experiments). **(I)** Representative images of F-actin/G-actin ratios in HeLa cells overexpressing LCP1 plasmids with/without inhibitors. This experiment was quantified by controlling for uniform loading amounts. **(J)** Statistical analysis of gray values from three independent experiments using ImageJ.

As a key actin cross-linker, LCP1 binds to and stabilizes parallel F-actin filaments.^13^ To investigate the functional consequences of the identified mutations, we first examined F-actin polymerization and LCP1 expression patterns in patient-derived cells. Confocal microscopy analysis revealed significantly increased F-actin and LCP1 staining intensities in both CD4^+^ T cells and neutrophils from P1 compared with HCs (Fig. 5A–D). Anti-CD3 stimulation induced greater LCP1 staining in P1's T cells, with an earlier peak time compared with HCs. Flow cytometry confirmed these findings in resting T cells, B cells, and neutrophils (Fig. S4A–S4D). Similarly, F-actin levels were elevated in L362F patient-iPSCs and LCP1^+/L362F^/^L362F/L362F^ iPSC-derived neutrophils versus controls (Figure 3, Figure 5E).

In 293T cells expressing comparable levels of WT, L362F, and A365D LCP1, protein degradation rates were similar after cycloheximide treatment (Fig. S4E). However, F-actin levels were significantly higher in L362F and A365D groups (Fig. 5F). Scratch assays revealed impaired wound healing in L362F and A365D HeLa cells (Fig. 5G and H). Notably, the F-actin/G-actin ratio increased significantly in L362F and A365D HeLa cells versus WT, which was reversed by LCP1 inhibitor Oroxylin A (Fig. 5I and J).^28^ These results demonstrate that L362F and A365D are gain-of-function mutations.

### LCP1 mutations alter the transcriptomic profile of bone marrow mononuclear cells

**Figure 6:**
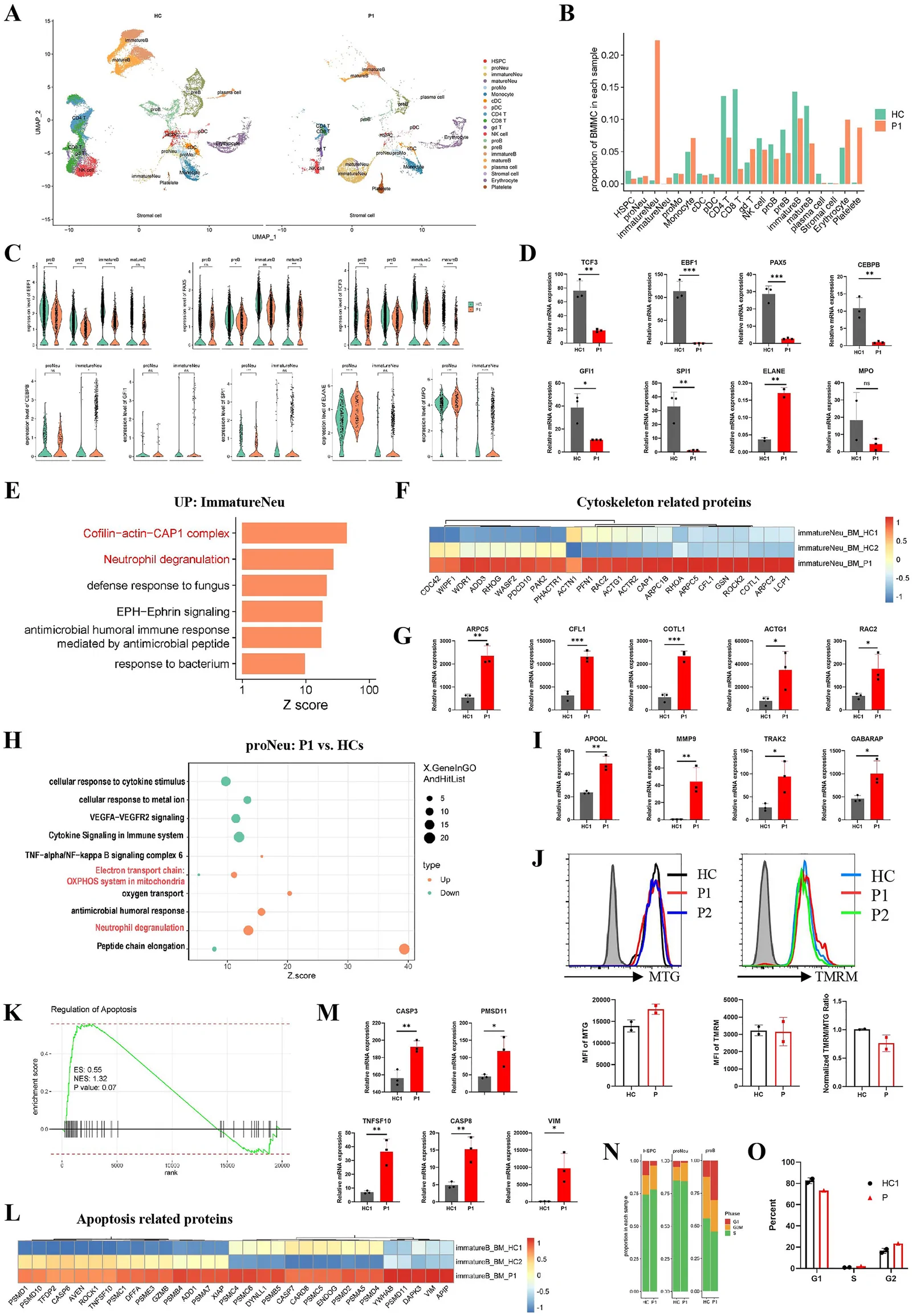
Single-cell RNA-sequencing analysis of bone marrow mononuclear cells (BMMCs) from P1. **(A)** UMAP visualization comparing P1 and healthy controls (HCs). After quality control, a total of 39,028 cells were analyzed. **(B)** Comparative percentages of BMMC populations between P1 and controls. **(C)** Differential expression analysis of transcription factors involved in B cell, neutrophil, and erythrocyte development. **(D)** Quantitative PCR quantification of the expression of transcription factors related to (C) in bone marrow RNA from P1 and HCs (*n* = 3). **(E)** GO enrichment analysis of differentially expressed genes in immature neutrophils. Pathways related to the cytoskeleton and neutrophil degranulation were significantly up-regulated in P1. **(F)** Heatmap displaying genes with ≥ 1.5-fold change between P1 and HCs; actin filament organization genes are highlighted (red: up-regulated; blue: down-regulated). A total of 24 significantly differential genes are shown. **(G)** Quantitative PCR validation of actin filament organization genes in bone marrow RNA from P1 and HCs (*n* ≥ 3). **(H)** Functional analysis of differentially expressed genes in proNeu. Pathways related to mitochondrial oxidative phosphorylation and neutrophil degranulation were significantly up-regulated in P1. **(I)** Quantitative PCR analysis of mitochondrial oxidative respiratory chain genes in bone marrow RNA from P1 and HCs (*n* ≥ 3). **(J)** Flow cytometric measurement of mitochondrial content (MTG) and reactive oxygen species (MitoSOX) in peripheral blood neutrophils from P1 and P2. The experiment was performed once, and the statistical analysis combined data from the patients. **(K)** Gene set enrichment analysis (GSEA) revealed significant enrichment of apoptosis-related pathways in immature B cells from patient P1, with most genes up-regulated and a few down-regulated in P1 (*P* = 0.07). **(L)** The heatmap represents the significantly up-regulated apoptosis-related differentially expressed genes in immature neutrophils from patient P1, comprising a total of 27 genes. **(M)** Quantitative PCR validation results of apoptosis-related genes analyzed in bone marrow RNA from P1 and HCs (*n* ≥ 3). **(N)** Cell cycle distribution of bone marrow progenitor cells from patient P1. Cell cycle phase assignment was based on module scores for S and G2M phase genes. **(O)** Flow cytometric analysis of the neutrophil cycle in patient P1 revealed a reduced proportion of cells in the G1 phase and a higher proportion in the G2 phase.

To investigate LCP1 dysfunction in hematopoiesis, we performed single-cell RNA-sequencing analysis of bone marrow mononuclear cells from P1 and two HCs. After quality control, 39,028 cells were classified into 20 distinct clusters (Fig. 6A; Fig. S5A). The bone marrow mononuclear cell analysis revealed a significant increase in immature neutrophils (immatureNeu), while neutrophil precursors (proNeu) showed no significant change (Fig. 6B). Key neutrophil differentiation factors (CEBPB, GFI1, SPI1) were down-regulated in proNeu and immatureNeu, whereas granule-associated genes (ELANE, MPO) were up-regulated in proNeu but down-regulated in immatureNeu (Fig. 6C and D), suggesting neutrophil differentiation defects. Both T and B cell compartments showed reduced proportions (Fig. 6B). Early B-cell development transcription factors (PAX5, EBF1, TCF3) were significantly down-regulated across B-cell subsets (Fig. 6C and D), consistent with flow cytometry findings of decreased peripheral neutrophils and B cells (Fig. 3A–C; Table 2). Bone marrow neutrophils of P1 exhibited reduced CD11b and CD16 expression (Fig. S5B),^29^ further indicating immaturity.

Functional enrichment analysis highlighted neutrophil degranulation and cytoskeletal gene up-regulat**i**on in immature neutrophils (Fig. 6E and F; Fig. 5C), confirmed by quantitative PCR (Fig. 6G). This aligned with elevated F-actin levels in P1's peripheral blood cells and iPSC-derived neutrophils. Mitochondrial function genes (including APOOL, MMP9, TRAK2, GABARAP) were up-regulated in proNeu (Fig. 6H) and other hematopoietic cells (Fig. 6I; Fig. S5E and S5F). Flow cytometry revealed increased mitochondrial mass and reduced membrane potential in P1's neutrophils (Fig. 6J; Fig. S5G), supporting LCP1's role in mitochondrial regulation.^30^^,^^31^ Cell cycle analysis showed G2/M-phase accumulation in neutrophils (Fig. 6N, O; Fig. S5H).

Apoptosis genes were enriched in immature B cells (Fig. 6K–M) and other marrow populations (Fig. S5I and S5J). Additional B-cell subsets showed enrichment in mitochondrial assembly, chromatin organization, and stress response genes (Fig. S5J–S5M).

In summary, transcriptomic profiling demonstrated LCP1 mutation-associated dysregulation in neutrophil/B-cell differentiation, apoptosis, mitochondrial function, and cell cycle pathways, correlating with observed phenotypes.

### LCP1^+/L362F^ mice phenocopy patient manifestations

**Figure 7:**
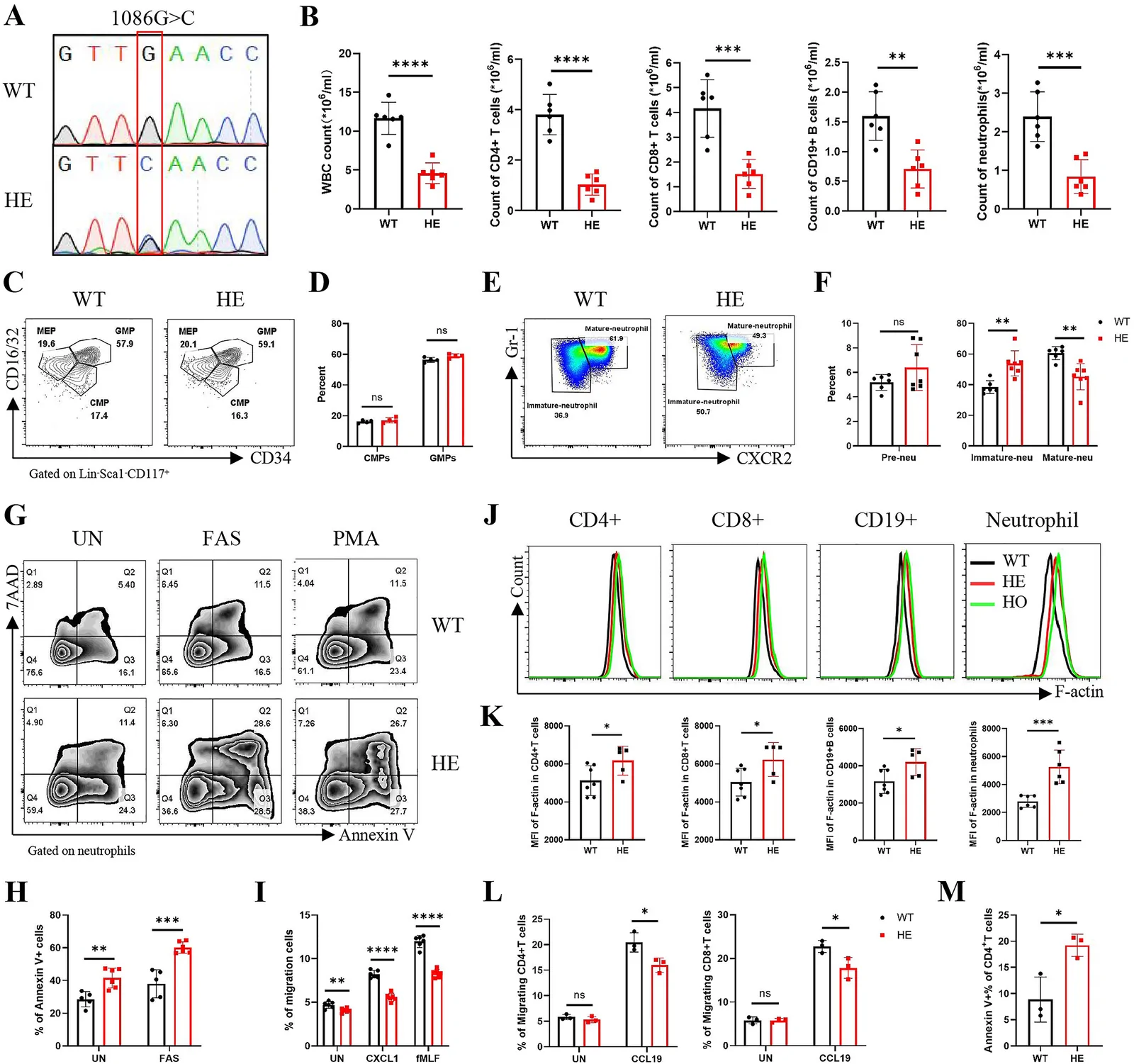
*LCP1*^+/L362F^ mice exhibit cytopenia, F-actin hyperpolymerization, increased neutrophil apoptosis, and impaired chemotaxis, recapitulating patient phenotypes. **(A)** Sanger sequencing confirmed LCP1 mutation sites (c.1086G>C). HE, heterozygous. **(B)** Peripheral blood lineage marker staining in LCP1^+/L362F^ mice (*n* = 6 per group). Top, from left: white blood cells (WBC), CD3^+^CD4^+^ T cells, CD3^+^CD8^+^ T cells, CD19^+^ B cells, and neutrophils (*P* values were calculated using Student's *t*-test). **(C)** Myeloid precursor profiles (common myeloid progenitors/CMPs and granulocyte-macrophage progenitors/GMPs) in WT and LCP1^+/L362F^ mice in bone marrows (*n* = 4). **(D)** CMP (CD16/32^low^CD34^+^) and GMP (CD16/32^high^CD34^+^) frequencies in BMCs (*P*-values were calculated using Student's *t*-test). **(E)** Neutrophil developmental stages in bone marrow: proNeu (Lin^–^Gr-1^+^CD11b^+^CXCR4^+^c-kit^int^CXCR2^–^), immature (Lin^–^Gr-1^+^CD11b^+^CXCR4^–/low^c-kit^low/–^CXCR2^–^), and mature (Lin^–^Gr-1^+^CD11b^+^CXCR4^–/low^c-kit^low/–^CXCR2^+^) neutrophils (*n* > 6). **(F)** Bone marrow proNeu, immature, and mature neutrophils from WT and LCP1^+/L362F^ mice were identified by noted markers. Two-way ANOVA with Sidak multiple comparison test was used to determine significance. **(G, H)** FAS- or PMA-induced early (Annexin V^+^7-AAD^–^) and late (Annexin V^+^7-AAD^+^) apoptosis in WT and LCP1^+/L362F^ mouse bone marrow neutrophils. The bar graph illustrates the proportion of Annexin V-positive cells in the UN (unstimulated) group and the FAS stimulation group. **(I)** Neutrophil migration assays in WT and LCP1^+/L362F^ mouse bone marrow neutrophils using 5 μm Transwell membranes. Spontaneous migration was assessed in unstimulated cells, while induced migration was measured after stimulation with 50 ng/mL CXCL-1 or 10 nmol/L fMLF. Data represent ≥ 3 independent experiments. Significance was determined by two-way ANOVA with Sidak's multiple comparison test. **(J)** F-actin expression in WT and LCP1^+/L362F^ mouse splenic lymphocytes and bone marrow neutrophils by flow cytometry (*n* > 5 per group). LCP1^+/L362F^ HO (homozygous) mice exhibit more elevated levels of F-actin expression. **(K)** F-actin mean fluorescence intensity (MFI) quantification in CD4^+^, CD8^+^, CD19^+^, and neutrophils (*P*-values were calculated using Student's *t*-test). **(L)** WT and LCP1^+/L362F^ mouse splenic CD4^+^ and CD8^+^ T cell migration with/without CCL19. Significance was determined by two-way ANOVA with Sidak's multiple comparison test. **(M)** Apoptosis of anti-CD3/CD28-stimulated CD4^+^ T cells after 3-day culture. The bar graph illustrates the proportion of Annexin V-positive cells (*P*-values were calculated using Student's *t*-test).

To establish the causal relationship between the LCP1 L362F mutation and the clinical phenotype observed in patients, we generated LCP1^+/L362F^ C57/BL6 mice using CRISPR/Cas9 (Fig. 7A; Fig. S6A). LCP1^+/L362F^ mice exhibited leukocytopenia, neutropenia, and lymphocytopenia (Fig. 7B), mirroring patient manifestations. While peripheral blood and bone marrow cellular composition ratios remained comparable to WT mice (Figs. S6B and S7A), we observed significant alterations in neutrophil development. Morphological and electron microscopy analyses revealed increased immature neutrophils in LCP1^+/L362F^ bone marrow (Fig. S6D and S6E). Flow cytometry demonstrated decreased mature neutrophils (Lin^–^Gr-1^+^CD11b^+^CXCR4^–/low^c-kit^low/–^CXCR2^+^) and increased immature neutrophils (Lin^–^Gr-1^+^CD11b^+^CXCR4^–/low^c-kit^low/–^CXCR2^–^) (Fig. 7E and F; Fig. S7C), despite normal common myeloid progenitor and granulocyte-macrophage progenitor populations (Fig. 7C and D; Fig. S7B). These findings suggest that LCP1 L362F impairs neutrophil maturation, consistent with patient-derived neutrophils. Additionally, LCP1^+/L362F^ neutrophils showed elevated apoptosis (Fig. 7G and H) and impaired chemotaxis (Fig. 7I), collectively contributing to peripheral neutropenia. F-actin levels were significantly increased in LCP1^+/L362F^ lymphocytes and neutrophils (Fig. 7J and K), while LCP1 phosphorylation at Ser 5 and total protein expression remained unchanged (Fig. S6F).^32^ Immunophenotypic analysis revealed normal T and B cell development in LCP1^+/L362F^ mice, with comparable cellular composition in lymphoid organs (Fig. S6C, S6G–S6J, S6O, S6P). Although T cell activation and cytokine production were unaffected (Fig. S6K–S6M), mutant mouse CD4^+^ T cells exhibited increased apoptosis, and both CD4^+^ and CD8^+^ T cells showed impaired migration to CCL19 (Fig. 7L and M), potentially explaining peripheral lymphocytopenia. Notably, the lymphocyte activation and senescence markers observed in patient P2 were not replicated in LCP1^+/L362F^ mice (Fig. S6J, S6N).^33^

These findings demonstrate that LCP1^+/L362F^ mice recapitulate key hematological abnormalities observed in patients, establishing genotype-phenotype causality in mice.

### Excessive F-actin aggregation enhances VDAC1 oligomerization, inducing apoptosis

**Figure 8:**
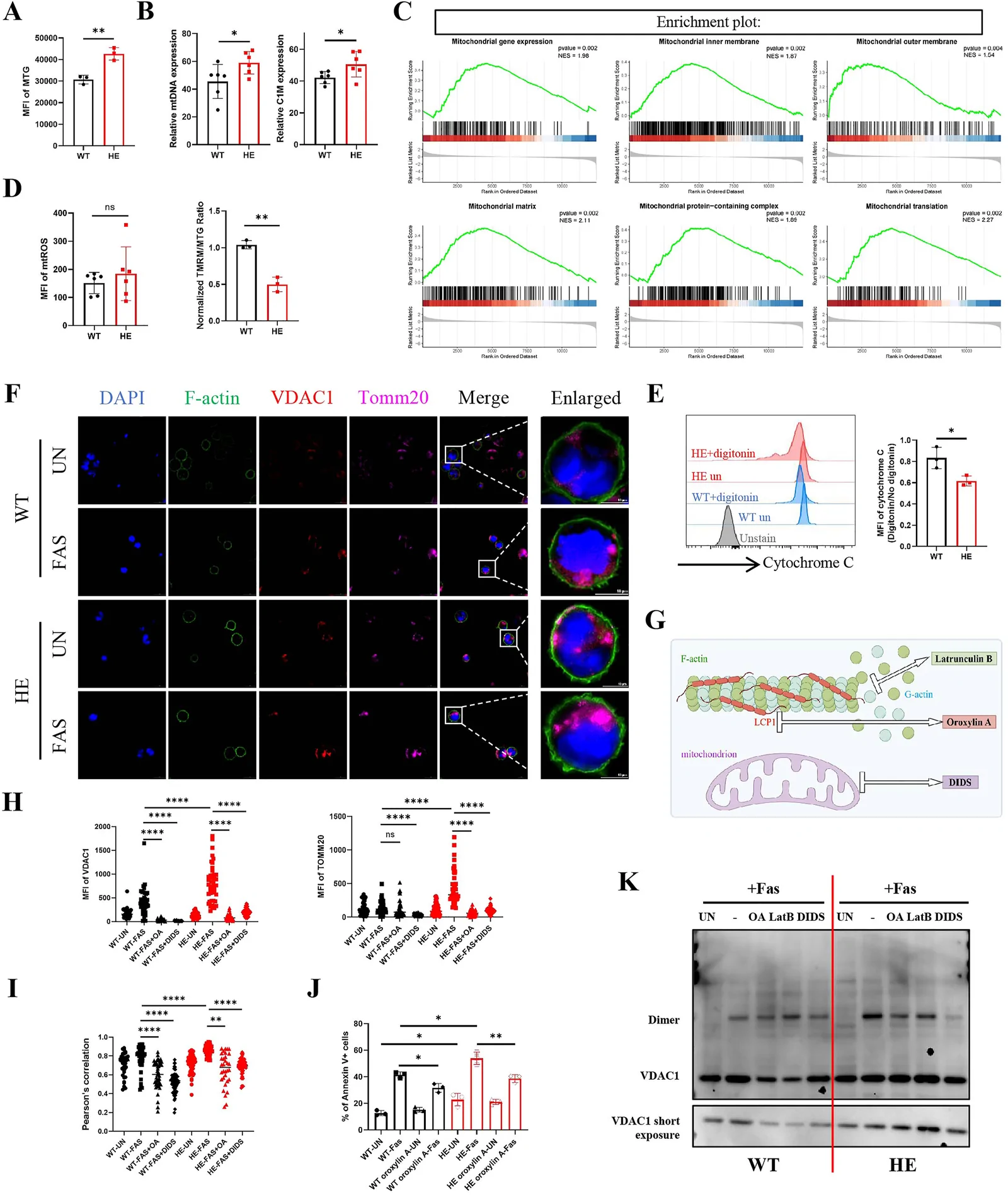
F-actin accumulation induces VDAC1 oligomerization on mitochondrial membranes, increasing inner membrane permeability and reducing mitochondrial membrane potential. **(A**–**D)** Mitochondrial parameter quantification in WT and LCP1^+/L362F^ bone marrow neutrophils. (A) MitoTracker Green (MTG) levels. (B) Mitochondrial DNA copy number, expressed as mt-DNA/C1M to nuclear DNA (B2M) ratio. (C) Gene set enrichment analysis (GSEA) of mitochondria-related pathways in WT and LCP1^+/L362F^ mouse bone marrow neutrophils. (D) Mitochondrial membrane potential (MMP, TMRM/MTG ratio) and mtROS levels. **(E)** Intracellular cytochrome C (Cyt C) staining in digitonin-permeabilized and non-permeabilized bone marrow neutrophils (*n* = 3). **(F)** Confocal microscopy of FAS-induced LCP1^+/L362F^ mouse bone marrow neutrophils stained for F-actin (phalloidin, green), VDAC1 (red), and Tomm20 (pink). The scale bar represents 10 μm, and the image on the right is digitally magnified. **(G)** Schematic of targeted inhibition strategies. Oroxylin A (LCP1 inhibitor), LatB (G-actin polymerization blocker), and DIDS (VDAC1 inhibitor). **(H)** Quantification of mean fluorescence intensity (MFI) for VDAC1 and Tomm20 from Figure 8F. Significance was determined by two-way ANOVA with Sidak's multiple comparison test. **(I)** VDAC1–Tomm20 colocalization by Pearson's coefficient. **(J)** WT and LCP1^+/L362F^ mouse bone marrow neutrophil apoptosis after inhibitor treatment. Three independent replicate experiments. **(K)** VDAC1 oligomerization by Western blotting in inhibitor-treated neutrophils. This shows one out of two independent replicate experimental results.

It is of great interest to investigate the underlying mechanism since LCP1 gain-of-function mutant neutrophils exhibited maturation defects, cell cycle arrest, impaired migration, and increased apoptosis. Single-cell RNA-sequencing analysis of patient bone marrow showed enrichment of mitochondrial oxidative phosphorylation, suggesting mitochondrial pathway-associated apoptosis. Mitochondrial mass was elevated in LCP1^+/L362F^ mice's bone marrow neutrophils, as demonstrated by increased MitoTracker Green (MTG) staining and mtDNA levels (Fig. 8A and B). Gene set enrichment analysis (GSEA) indicated enrichment of mitochondrial biogenesis and membrane-related genes in LCP1^+/L362F^ groups (Fig. 8C). The tetramethylrhodamine methyl ester (TMRM)/MTG ratio, reflecting mitochondrial membrane potential per unit mass, decreased in patients and mutant mice, while mtROS levels remained stable (Figure 6, Figure 8D). Digitonin-treated LCP1^+/L362F^ neutrophils showed preferential cytochrome C (Cyt C) loss,^34^ indicating heightened mitochondrial membrane permeability (Fig. 8E). These findings were corroborated in patient-derived iPSC-neutrophils (Fig. S2H–S2J). To investigate how LCP1 gain-of-function-induced F-actin aggregation causes mitochondrial dysfunction, we examined its interaction with VDAC1, a mitochondrial outer membrane channel protein.^35^, ^36^, ^37^, ^38^, ^39^, ^40^ F-actin colocalized partially with Tomm20 and VDAC1 in LCP1^+/L362F^ iPSC-neutrophils (Fig. S8A). FAS induction increased apoptosis in LCP1^+/L362F^ neutrophils versus WT (Fig. 7G and H), alongside elevated VDAC1–Tomm20 colocalization, suggesting F-actin-VDAC1 interaction (Fig. 8F–H). To confirm causality, we used inhibitors: Oroxylin A (LCP1 inhibitor),^28^ LatB (G-actin polymerization blocker),^41^ and DIDS (VDAC1 inhibitor) (Fig. 8I).^42^ Oroxylin A and LatB reduced F-actin assembly and partially rescued apoptosis post-FAS stimulation (Fig. 8J; Fig. S8B). Inhibitor treatment also diminished VDAC1–Tomm20 colocalization and VDAC1 oligomerization, a marker of apoptosis, as shown by confocal microscopy and Western blotting (Fig. 8F, G, K). Thus, excessive F-actin aggregation promotes apoptosis by enhancing VDAC1 oligomerization, increasing mitochondrial inner membrane permeability, and reducing membrane potential.

## Discussion

Actin, a key cytoskeletal component, polymerizes into F-actin and forms networks through actin-binding proteins.^10^^,^^11^ Its dynamic properties are tightly regulated by these proteins. Genetic mutations affecting actin regulatory proteins lead to immuno-actinopathies, a group of primary immunodeficiency.^5^^,^^6^ These conditions manifest diverse innate and adaptive immune deficiencies, directly or indirectly disrupting actin polymerization/depolymerization dynamics and network organization (Table S3)^7^, ^8^, ^9^^,^^43^, ^44^, ^45^, ^46^, ^47^, ^48^, ^49^, ^50^, ^51^, ^52^, ^53^, ^54^, ^55^, ^56^. Loss-of-function mutations primarily impair actin polymerization, resulting in neutrophil chemotaxis/migration defects, lymphocytopenia, and thrombocytopenia.^9^^,^^43^^,^^44^ Conversely, gain-of-function mutations in Wiskott-Aldrich syndrome protein (WASp) and Rac family small GTPase 2 (RAC2) enhance actin polymerization, causing neutropenia, neutrophil migration defects, variable lymphocytopenia, and increased apoptosis.^45^^,^^46^^,^^54^^,^^55^ This study identified three patients from two families with heterozygous LCP1 gain-of-function mutations (L362F and A365D) presenting congenital neutropenia and variable combined immunodeficiency.

Mahat et al reported a severe neutropenia case with LCP1 I232F mutation.^17^ Studies using HeLa and 32D myeloblast cell lines overexpressing LCP1 I232F demonstrated increased F-actin, cell cycle arrest, dysplasia, and impaired proliferation and migration. The patient exhibited bone marrow cell apoptosis and CD3^+^CD8^+^ T lymphocytopenia. Although not classified as inborn errors of immunity in IUIS,^25^ Mahat's findings offer genetic clues for our cases. Recently, Hernandez et al described a family with three members carrying an LCP1 splice-site mutation, presenting recurrent infections (otitis media, tonsillitis) and hematologic abnormalities (leukopenia, lymphopenia, neutropenia, mild thrombocytopenia).^57^ Functional studies revealed that this variant exerted dominant-negative effects (loss-of-function), impairing lymphocyte migration and cytokinesis. Unlike these cases, our patients show neutropenia as well as combined T/B cell immunodeficiency but normal platelet counts. We provide novel evidence of neutrophil/lymphocyte apoptosis and validated neutrophil differentiation defects using patient-derived iPSCs. Notably, F-actin elevation was observed in primary neutrophils, lymphocytes, iPSCs, and L362F/A365D-expressing cell lines, indicating LCP1 dysfunction. Through cell line models and inhibitor studies, we confirmed the functional consequences of LCP1 L362F and A365D mutations. Therefore, we refer to this immunodeficiency syndrome caused by LCP1 gain-of-function mutations as activated LCP1-associated immunodeficiency syndrome.

The three patients carry heterozygous LCP1 mutations, indicating autosomal dominant inheritance. Our investigation of family B was limited, particularly regarding P3's father's clinical data during childhood and adolescence, which were unavailable for follow-up. He experienced transient leukopenia and neutropenia during childhood, along with a history of frequent oral ulcers, although he has no history of severe infections. This appears to indicate that LCP1 mutations exhibit incomplete penetrance, with current data suggesting that this may stem from the mutation's relatively weak impact on the cytoskeleton—specifically, the A365D mutation induces a smaller increase in the F-actin/G-actin ratio compared with the L362F mutation.^58^ Recently, the genetic study of French patients with undiagnosed chronic neutropenia by Marti et al identified 5 affected individuals with heterozygous LCP1 Ser170Leu mutations.^59^ These patients consistently exhibited chronic neutropenia with intermittent lymphopenia of varying severity, supporting our findings, though the study lacked mechanistic investigation and knock-in model confirmation. During the revision of our manuscript, van Bergen and colleagues reported four families carrying heterozygous LCP1 mutations, similarly demonstrating incomplete penetrance.^60^ Their work aligns closely with our findings in multiple aspects, including the observation that LCP1 mutations lead to excessive F-actin aggregation, and the demonstration through iPSC-based differentiation that LCP1 mutations disrupt myeloid hematopoiesis and cause cell cycle defects. Together, our findings and those of Mahat, Hernandez, Marti, and van Bergen collectively establish that autosomal dominant LCP1 mutations cause a hematopoietic disorder characterized primarily by neutropenia, lymphocytopenia, and hypogammaglobulinemia. Finally, we propose possible reasons for the incomplete penetrance of LCP1 mutations, such as the pathogenic strength of the mutation site, variable protein expression, epigenetic and environmental factors, as well as the influence of other unknown genetic factors.

The observation that LCP1^+/L362F^ knock-in mice recapitulated key clinical manifestations in patients, including absolute neutrophil count reduction in the peripheral blood, was unexpected. Most severe congenital neutropenia-associated genes do not induce neutropenic phenotypes in murine models.^61^^,^^62^ Analysis showed that bone marrow neutrophil counts and peripheral blood neutrophil percentages were unchanged in LCP1 mutant mice, but these animals displayed increased immature myeloid precursors, enhanced apoptosis, and impaired cytokine-directed neutrophil migration. These pathological changes likely explain the peripheral neutropenia. L362F patient-iPSCs and gene-edited L362F heterozygous/homozygous iPSCs all showed impaired neutrophil differentiation, increased F-actin aggregation, and excessive apoptosis *in vitro*. Single-cell transcriptome analysis confirmed bone marrow neutrophil maturation arrest. These two models together demonstrate LCP1 as the causative gene.

Our study preliminarily elucidates the mechanisms of neutropenia and leukocytopenia induced by L362F and A365D mutations. LCP1 regulates actin cytoskeleton dynamics, which impacts mitochondrial metabolism, signaling pathways, cell cycle progression, and migration, thereby influencing immune cell fate decisions between differentiation and apoptosis. Compared with activated LCP1-associated immunodeficiency syndrome, other actinopathies, including XLN (WAS gain-of-function mutation), RAC2 (gain-of-function), STK4 deficiency, and WDR1 deficiency, have been more extensively characterized, revealing shared pathogenic mechanisms.^34^, ^35^, ^36^, ^37^, ^38^^,^^43^, ^44^, ^45^ Growing evidence establishes actin cytoskeleton and actin-binding proteins as key regulators of mitochondrial dynamics, biogenesis, and apoptosis.^37^^,^^38^^,^^63^^,^^64^ Single-cell transcriptomic analysis of bone marrow mononuclear cells from patient P1 and RNA-sequencing data from LCP1^+/L362F^ mouse neutrophils showed elevated expression of oxidative phosphorylation-related genes in immature neutrophils. Flow cytometry confirmed mitochondrial dysfunction through altered mitochondrial mass and membrane potential (ΔΨm). Mechanistically, LCP1 gain-of-function-induced F-actin hyperaggregation promotes VDAC1 oligomerization (validated by SPR-measured VDAC-actin binding^40^), compromising mitochondrial inner membrane integrity (increased permeability and ΔΨm collapse) and triggering apoptosis.^36^, ^37^, ^38^, ^39^ Although our data indicate that LCP1 mutations stabilize F-actin networks by inhibiting depolymerization, the exact regulatory mechanism requires further investigation. Notably, Lucas et al recently demonstrated LCP1 functions as a pH-sensitive actin-bundling sensor,^65^ with mutants showing altered pH responsiveness that affects cytoskeletal remodeling—a finding that broadens our understanding of actin-related pathologies.

Regarding long-term follow-up and prognosis in our patient cohort: among the three cases, none required hematopoietic stem cell transplantation, with P2 surviving to adulthood. Treatment protocols included supportive care, antibiotic prophylaxis, and G-CSF administration to maintain neutrophil counts during acute infections. Notably, no patients developed myelodysplastic syndrome or acute myeloid leukemia during follow-up, though long-term G-CSF use warrants continued surveillance. Potential therapeutic candidates include LCP1-specific inhibitors or actin polymerization blockers. *In vitro* studies demonstrated Oroxylin A's efficacy in suppressing LCP1 mutant-induced F-actin elevation, supporting its therapeutic potential. Further screening of LCP1-specific inhibitors is recommended. The efficacy of regular intravenous immunoglobulin and anti-allergy therapies requires further investigation.

This study identified LCP1 gain-of-function mutations causing a primary immunodeficiency disorder, characterized by congenital neutropenia and combined immunodeficiency. Using patient-derived cells, LCP1-mutant cell lines, iPSCs, and mouse models, we demonstrated actin dysregulation mechanisms, revealing LCP1-regulated F-actin polymerization abnormalities. These models enable molecular mechanism investigation and therapeutic target identification.

## CRediT authorship contribution statement

**Lang Yu:** Writing – original draft, Visualization, Validation, Methodology, Investigation, Data curation. **Bo Zhou:** Visualization, Validation, Investigation, Data curation. **Wei Liu:** Writing – review & editing, Resources, Conceptualization. **Wenhui Li:** Investigation, Data curation. **Qinglv Wei:** Visualization, Investigation, Data curation. **Yishi Zhang:** Validation, Investigation. **Chen Li:** Validation, Investigation. **Wendao Li:** Validation, Investigation. **Yulin Li:** Validation, Investigation. **Guangzhao Li:** Validation, Investigation. **Gan Sun:** Validation, Investigation. **Rui Gan:** Validation, Investigation. **Ran Chen:** Validation, Investigation. **Wenjing Zhang:** Validation, Investigation. **Anle Zeng:** Validation, Investigation. **Rongtao Zhao:** Validation, Investigation. **Wenli He:** Validation, Investigation. **Yanjun Jia:** Writing – review & editing, Validation, Methodology, Investigation, Funding acquisition. **Lina Zhou:** Writing – original draft, Validation, Methodology, Investigation. **Zhiyong Zhang:** Writing – original draft, Methodology, Investigation. **Xuemei Tang:** Writing – review & editing, Resources, Methodology, Data curation. **Xiang Qiu:** Writing – review & editing, Supervision, Investigation, Data curation. **Qing Zhou:** Writing – review & editing, Validation, Formal analysis. **Wenxia Song:** Writing – review & editing, Supervision, Formal analysis. **Xiaodong Zhao:** Writing – review & editing, Supervision, Resources, Project administration, Funding acquisition, Formal analysis, Conceptualization. **Yunfei An:** Writing – review & editing, Supervision, Project administration, Funding acquisition, Formal analysis, Conceptualization.

## Ethics declaration

The study was performed following the Declaration of Helsinki and approved by the Ethics Committee of Children's Hospital of Chongqing Medical University (20210331, Chongqing, China). Written informed consent for participation in this study was obtained from the patients' parents. All animal work was reviewed and approved by the Institutional Animal Care and Usage Committee of Children's Hospital of Chongqing Medical University.

## Data availability

Raw sequencing reads for single-cell RNA-sequencing data are deposited in the SRA database: https://www.ncbi.nlm.nih.gov/geo/query/acc.cgi acc = GSE252973. For review of record GSE252973 while it remains in private status: ilonisskpfkzlep. For mouse RNA-sequencing data, see https://dataview.ncbi.nlm.nih.gov/object/PRJNA1247919. Other data and materials can be obtained from the corresponding author through reasonable requests.

## Funding

This work was supported by the Chongqing Medical Scientific Research Project (China) (No. 2024GGXM004) (Joint project of Chongqing Health Commission and Science and Technology Bureau of China), Chongqing Natural Science Foundation Innovation and Development Joint Fund (China) (No. CSTB2024NSCOLZX0100), Clinical Research Project of Children's Hospital of Chongqing Medical University (No. CHCMU-2024-XKDF-1001), Graduate Mentor Team Program of Chongqing Municipal Education Commission of China (No. 2019-09-66), and the 10.13039/501100001809National Natural Science Foundation of China (No. 82070135, 82271772).

## Conflict of interests

Xiaodong Zhao is an Editorial Board member/deputy editor-in-chief for *Genes & Diseases* and was not involved in the editorial review or the decision to publish this article. The remaining authors declared no competing interests.
