# Supplementary material for: Autosomal dominant gain-of-function mutations in LCP1 cause a syndromic neutropenia and immunodeficiency
Source: Genes Dis. 2026 May 12;14(1):102232. doi: 10.1016/j.gendis.2026.102232 (PMC13589076; doi:10.1016/j.gendis.2026.102232)
Supplement: Supplementary file 1 [file mmc1.doc]

**Supplementary materials for**

**Autosomal dominant gain-of-function mutations in LCP1 cause a novel syndromic neutropenia and immunodeficiency**

**Supplementary Text**

**Detailed clinical presentation of P2 and P3**

Family A

Since age 2, P1 has exhibited recurrent allergic manifestations, including post-infectious urticaria (particularly following respiratory infections), egg hypersensitivity, and episodic epistaxis. Laboratory evaluation during allergic flares demonstrated elevated type 2 inflammatory markers, notably IgE and eosinophilia (Table 2 and Supplementary Table 1). Flow cytometric analysis revealed a modest Th2 cell skewing in P1 (Supplementary Figure 3G), though without concomitant elevation of characteristic Th2 cytokines (IL-4, IL-5, and IL-13), suggesting these allergic responses may be primarily mediated through mast cell and eosinophil pathways rather than classical Th2 immunity.

P2 has experienced recurrent pneumonia and otitis media since childhood, though early medical records are unavailable. His clinical course includes sensorineural hearing loss requiring hearing aids at age 18, pulmonary tuberculosis necessitating quadruple antitubercular therapy at 23, and hepatitis B infection diagnosed at 26. Additionally, he presents with chronic mucocutaneous features including recurrent oral ulcerations, persistent gingivitis, impaired wound healing, and atopic manifestations (eczema and atopic dermatitis). Currently age 34 with stable condition at our center's diagnosis, he maintains good overall health status despite radiographic evidence of mild bronchiectasis. No other Family A members exhibit clinical symptoms or harbor the LCP1 L362F variant.

Family B

Due to loss to follow-up, detailed clinical records for P3's father (who carries the same LCP1 variant as P3) are unavailable for review. He experienced transient leukopenia and neutropenia during childhood, along with a history of frequent oral ulcers, although he has no history of severe infections. His current health status appears favorable, with no reported recurrent infections or other clinical manifestations. Routine hematologic testing has not detected neutropenia or lymphopenia. Study participation was limited to provision of blood samples for genetic sequencing, precluding collection of adequate material for functional validation assays. Given these constraints in both historical clinical documentation and available biospecimens, we have designated his case as having uncertain or a mild clinical significance.

**Supplementary Methods**

**Isolation of Mononuclear Cells and Neutrophils from Human Peripheral Blood**

Heparinised peripheral blood samples underwent centrifugation (700 g, 5 min, at room temperature) to separate plasma from peripheral blood cells. Cells were then diluted 1:1 in phosphate-buffered saline (PBS), layered onto Ficoll-Paque Premium (GE Healthcare, Sweden), and centrifuged at room temperature (800 g, 20 min) to isolate peripheral blood mononuclear cells (PBMCs). Neutrophils were separated from other cells via dextran deposition and hypotonic red blood cell lysis, and subsequently cultured in Krebs–Ringer phosphate buffer.

**Neutrophils respiratory burst**

Respiratory burst activity was assessed by flow cytometry using dihydrorhodamine-1,2,3 (DHR) (Sigma-Aldrich) following established protocols^[1]^. Peripheral blood samples from both patients and healthy controls (HCs) were aliquoted into two 50 µl reactions. For stimulated conditions, samples were incubated with 18 µl of 2 ng/ml phorbol 12-myristate 13-acetate (PMA, Sigma-Aldrich) and 6 µl of 40 mmol DHR, while unstimulated control samples received 18 µl PBS and 6 µl of 40 mmol DHR, with both conditions maintained at 37°C for 20 minutes. Following red blood cell lysis performed according to standard protocols, cells were resuspended in 300 µl PBS for subsequent flow cytometric analysis. The Neutrophil Oxidative Index (NOI) was determined by calculating the ratio of mean fluorescence intensity (MFI) in PMA-stimulated samples to that in unstimulated control samples.

**Reactive Oxygen Species (ROS) Analysis**

Neutrophil endogenous and exogenous ROS levels were assessed using luminol and isoluminol chemiluminescence (CL) assays in a Synergy H1 multi-mode Reader (BioTek, USA), as previously outlined^[2]^. In brief, 3×105 cells/ml neutrophils were stimulated with various reagents in 0.2 ml reactions in white 96-well plates (Costar). Intracellular ROS production was monitored in the presence of 20 µmol luminol (Sigma), 50 U/ml SOD (Solaibio), and extracellular ROS in the presence of 20 µmol isoluminol (Sigma) and 4 U/ml HRP (Calbiochem). CL values are presented as relative light units (RLU).

**CDR3 Spectratyping**

Spectratyping analysis of CDR3 involved the use of 23 primers designed to amplify T cell-specific receptors, along with a 5' FAM-labeled CB probe. The PCR products were dispatched to Sangong Biotech (Shanghai, China) for sequencing. Data analysis was conducted using GeneMapper v3.5, and TCRVβ diversity was evaluated employing a scoring system (complexity score = (sum of all peak heights / sum of major peak heights) × number of major peaks). Major peaks were defined as those with amplitudes of at least 10% of the sum of all peak heights^[3]^.

**Flow Cytometry Analysis**

For Tfh, Tfr, and Th1/2/17 cell analysis, a total of 250 µl of peripheral blood from both patients and healthy controls was processed and evenly distributed into five flow tubes. Tubes were designated for Tfh, Tfr, and Th1/2/17 cell subsets. Following staining with corresponding markers, flow cytometry was employed to detect CD4^+^ cell subsets.

| **Marker** | **Fluorescence Channel** | **Clone number** | **Article number** | **company** |
| --- | --- | --- | --- | --- |
| CD3 | Percp | HIT3a | 300326 | Biolegend |
| CD4 | PE-cy7 | RPA-T4 | 300512 | Biolegend |
| CD45RA | FITC | HI100 | 555488 | BD |
| CD45RO | APC | UCHL1 | 559865 | BD |
| CXCR5 | BV421 | J252D4 | 356920 | Biolegend |
| PD-1 | FTIC | EH12.2H7 | 329904 | Biolegend |
| ICOS (CD278) | PE | DX29 | 557802 | BD |
| CD127 | PE | A019D5 | 351304 | Biolegend |
| CD25 | APC | BC96 | 302610 | Biolegend |
| CXCR3 | APC | 1C6 | 561732 | BD |
| CCR6 (CD196) | PE | G034E3 | 353410 | Biolegend |

For regulatory T cell subsets analysis, 1×10^6^ PBMCs from patients and HCs were labeled with cell surface markers CD4, CD25 and CD45RA. Subsequently, the cells were fixed and permeabilized, and intracellular FOXP3, Helios, CD152 (CTLA-4) were labeled for detection and analysis using flow cytometry (all from Biolegend). For NK cell function, 1×10^6^ PBMCs were divided into experimental and control groups. K562 cells were added to the experimental group for co-incubation with PBMCs, while the control group received an equivalent volume of medium for co-incubation over a 4-hour period at 37℃ and 5% CO2. Subsequently, both the experimental and control groups were further divided into two parts, with and without CD107α. At 4℃, samples were kept away from light for 30 minutes before being detected and analyzed via flow cytometry. For CD4^+^ T cells IL-17A/IFN-γ production analysis, 1×10^6^ PBMCs were stimulated with CD3/28 or PMA/ionomycin for 72h. For T cells surface CD69, CD25 and PD-1, 1×10^6^ PBMCs were stimulated with CD3/28 for 48h. For quantification of intracellular F-actin and LCP1, cells were fixed and permeabilized by using 0.2% Triton-X and probed by using Phalloidin-iFluor 488 (1:1000) (Abcam) or LCP1 antibody (Santa Cruz).Flow cytometry analysis of patient bone marrow cells was performed as follows: whole bone marrow cells were lysed to remove red blood cells, followed by staining with CD11b and CD16 antibodies. Analysis was conducted under a live/dead (L/D) negative gate to exclude dead cells.

**Plasmids**

Full-length wild-type (WT) LCP1 cDNA was obtained from Youbao Biotechnology, Changsha, China. Mutant LCP1 cDNA was constructed by PCR mutagenesis and subcloned

into the 7.1-pCMV-3×Flag vector. The sequence is sequenced for confirmation by Sangon Biotech (Shanghai, China).

**Mitochondrial content, membrane potential, and reactive oxygen species (ROS) detection**

1×10^6^ human peripheral blood neutrophils (iPSc derived neutrophils or mice neutrophil) were incubated with a 1:2000 dilution of live/dead dye for 10 minutes. Mitochondrial content was labelled using MTG (Mitotracker Green), membrane potential was assessed with TMRM, and ROS levels were determined using MitoSOX (mitochondrial reactive oxygen species). For ROS quantification, cells were stimulated with PMA for 10 minutes before labelling.

**Flow cytometric analysis of mice**

For flow cytometric analysis of mouse peripheral blood. Single-cell suspensions of peripheral blood were obtained as previously described^[4]^. Cells were stained with 1:100 dilution of fluorescently conjugated antibodies against mouse CD19, CD3e, CD4, CD8, CD11b and Ly6G (all from Biolegend). Cells were collected on an BD celesta and data were analyzed using FlowJo. Cell numbers were quantified using phycoerythrin-conjugated fluorescent counting beads. For intracellular cytochrome C staining, bone marrow cells were first treated on ice for 8 min with either control or 6 μg/ml digitonin, then washed twice with PBS and labeled with antibodies for CD11b and Ly6G. Cells were then fixed and permeated with BM Cytofix Buffer sets (BD Biosciences) and stained with anti‐cytochrome C (Biolegend). For other flow cytometry methods used in mouse phenotyping, the experiments were performed according to previously established protocols^36,37^.
**Analysis of Neutrophil Subsets in Mouse Bone Marrow**

Neutrophils were isolated from mouse bone marrow using Histopaque gradient separation as described^[5]^. BM cells were isolated from mice, blocked with Fc receptor blocker (anti-mouse CD16/32 antibody, clone 93), and stained at 4℃ with various antibodies. Lineage markers for mouse BM neutrophil maturation study include anti-mouse CD3, B220, CD115, NK1.1, and SiglecF (Biolegend); the other anti-mouse antibodies include CXCR2, CXCR4, c-Kit, Gr-1, CD11b, CD16/32, CD34, Sca1 (all from Biolegend). CMPs were identified as CD16/32^low^CD34^+^, GMPs were identified as CD16/32^high^CD34^+^, Preneutrophils were identified as Lin^-^Gr-1^+^CD11b^+^CXCR4^+^c-kit^int^CXCR2^-^, immature neutrophils were identified as Lin^-^Gr-1^+^CD11b^+^CXCR4^-/low^c-kit^low/-^CXCR2^-^, and mature neutrophils were identified as Lin^-^Gr-1^+^CD11b^+^CXCR4^-/low^c-kit^low/-^CXCR2^+ [6,7]^.

**Supplementary Tables**

**Supplementary Table 1. The long-term follow-up results of the patient's peripheral blood routine tests.**

|  | **P1** | | | | | | | | **P2** | | | | | | **P3** | | | | | | |
| --- | --- | --- | --- | --- | --- | --- | --- | --- | --- | --- | --- | --- | --- | --- | --- | --- | --- | --- | --- | --- | --- |
| **Age at test** | 18d | 14m | 14m | 18m | 2y | 3y | 3y | Ref. values | 18y | 22y | 23y | 29y | 34y | Ref. values | 7m | 9m | 9m | 15m | 3y | 3y | Ref. values |
| **G-CSF** | No | No | Yes | No | No | No | Yes |  | No | No | No | No | No |  | No | No | Yes | No | No | Yes  Yes |  |
| **WBC count×10^3^/uL** | 0.28 | 0.1430 | 4.260 | 2.400 | 1.600 | 2.000 | 3.700 | 4.4-12.0 | 2.110 | 1.880 | 1.300 | 1.070 | 1.780 | 4.0-10.0 | 3.120 | 2.220 | 5.050 | 3.000 | 4.4 | 7.5 | 5.0-14.2 |
| **RBC count×10^3^/uL** | 4.810 | 4.290 | 4.620 | 4.730 | 4.720 | 4.050 | 4.500 | 4.1-5.3 | 4.840 | 4.170 | 3.990 | 3.640 | 4.050 | 3.5-5.5 | 4.240 | 3.830 | 3.880 | 4.110 | 4.45 | 4.48 | 4.1-5.5 |
| **Hemoglobin (g/dL)** | 158 | 115 | 129 | 129 | 126 | 108 | 122 | 104-151 | 120 | 113 | 106 | 97 | 139 | 110-160 | 83 | 76 | 85 | 85 | 108 | 96 | 103-138 |
| **Platelets×10^3^/uL** | 359 | 168 | 262 | 247 | 232 | 169 | 280 | 100-300 | 241 | 252 | 347 | 301 | 268 | 100-300 | 405 | 366 | 358 | 580 | 448 | 520 | 172-605 |
| **MPV (fl)** | 12.3 | 11.2 | 10.9 | 9.6 | 10.1 | 9.8 | 10.6 | 9-12.5 | 9.7 | 10.1 | 10.4 | 9.8 | 9.2 | 7.0-11.0 | 9.1 | 8.2 | 9.5 | 11.2 | 9..7 | 11.6 | 9-17 |
| **Lymphocyte×10^3^/uL** | 0.1410 | 0.930 | 1.660 | 1.510 | 1.010 | 0.530 | 0.440 | 1.6-9.0 | 0.530 | 0.480 | 0.360 | 0.180 | 0.690 | 1.0-4.0 | 2.780 | 1.860 | 2.060 | 2.210 | 4.0 | 3.6 | 2.8-10.0 |
| **Neutrophil×10^3^/uL** | 0.450 | 0.350 | 2.340 | 0.630 | 0.250 | 0.450 | 2.800 | 0.8-6.0 | 1.170 | 1.040 | 0.810 | 0.670 | 0.780 | 2.0-7.0 | 0.100 | 0.070 | 2.500 | 0.450 | 0.08 | 3.4 | 1.2-6.5 |
| **Monocytes×10^3^/uL** | 0.770 | 0.120 | 0.160 | 0.160 | 0.170 | 0.490 | 0.010 | 0.22-0.84 | 0.390 | 0.320 | 0.100 | 0.110 | 0.200 | 0.12-0.8 | 0.130 | 0.060 | 0.150 | 0.090 | 0.16 | 0.18 | 0.15-1.24 |
| **Eosinophils×10^3^/uL** | 0.170 | 0.020 | 0.090 | 0.100 | 0.860 | 0.480 | 0.250 | 0-0.45 | 0.010 | 0.030 | 0.060 | 0.100 | 0.050 | 0.02-0.5 | 0.100 | 0.230 | 0.250 | 0.230 | 0.15 | 0.21 | 0.06-1.22 |

**WBC, white blood cell. RBC, red blood cell. MPV, mean platelet volume.**

**Supplementary Table 2. Prediction of LCP1 mutation pathogenicity**

|  | **LCP1 c.1086G>C(L362F)** | | **LCP1 c.1094C>A(A365D)** | |
| --- | --- | --- | --- | --- |
| **Algorithm** | score | prediction | score | prediction |
| **SIFT** | 0.001 | Damaging | 0.0 | Damaging |
| **Polyphen-2 HDIV** | 1.0 | Probably_damaging | 1.0 | Probably_damaging |
| **Polyphen-2 HVAR** | 1.0 | Probably_damaging | 1.0 | Probably_damaging |
| **REVEL** | 0.846 | Damaging | 0.977 | Damaging |
| **Mutation Taster** | 1.0 | Disease_causing | 1.0 | Disease_causing |
| **PROVEAN** | -3.8 | Damaging | -5.75 | Damaging |
| **GERP++** | 5.34 | Conserved | 5.32 | Conserved |
| **CADD** | 25.6 | Damaging | 32 | Damaging |
| **Alphamissense** | 0.9977 | Pathogenic | 0.9998 | Pathogenic |

**Supplementary Table 3. Clinical and immunological characteristics of human inborn errors of immunity caused by actin cytoskeleton defects**

| **Gene defect** | **Gene or Protein function** | **Mutation type** | **Immune cell phenotypes** | **Clinical features** | **References** |
| --- | --- | --- | --- | --- | --- |
| **ACTB** | Non-muscle actin isoform | AR, LoF | Impaired chemotaxis and ROS production of neutrophils | Recurrent infections and mental disability | [8] |
| **WAS** | Nucleation factor for the  ARP2/3 complex | XR, LoF | Thrombocytopenia and lymphocytopenia | Recurrent infections, severe bleeding, eczema, autoimmune diseases, lymphoma | [9] |
|  |  | XR, GoF | Neutropenia, monocytopenia, thrombocytopenia, increased F-actin and apoptosis | Recurrent infections, malignancies | [10,11] |
| **MKL1** | Co-activator of SRF | AR, LoF | Reduced actin content and impaired actin polymerization, impaired migration, impaired spreading of neutrophils | Severe, recurrent bacterial infections | [12,13] |
| **ARPC1B** | Component of the ARP2/3 complex | AR, LoF | Thrombocytopenia, lymphocytopenia, and eosinophilia, impaired actin polymerization, impaired motility | Viral and bacterial infections, bleeding tendency, vasculitis, eczema, allergy | [14] |
| **ARPC5** | Component of the ARP2/3 complex | AR, LoF | Thrombocytopenia, decreased number of T regulatory cells, impaired cell migration, poor wound healing | Recurrent and severe infections, early-onset autoimmunity, inflammation, and dysmorphisms | [15] |
| **NCKAP1L** | Component of the WRC | AR, LoF | Lymphocytopenia, impaired actin polymerization, synapse formation, and immune cell migration | Recurrent bacterial and viral infections and atopic and allergic disease | [16,17] |
| **WIPF1** | Interactor of WASP and  stable and inactive WASP | AR, LoF | Thrombocytopenia, lymphocytopenia | Recurrent infections, eczema, papulovesicular/ulcerative lesions, bloody diarrhea | [18] |
| **CORO1A** | Actin-binding protein,  interacts with the ARP2/3  complex | AR, LoF | T-lymphocytopenia, increased F-actin, Very short telomere length | Recurrent bacterial and viral respiratory infections, skin lesions, chronic warts | [19] |
| **WDR1** | Enhances severing and  disassembly of actin  filaments by cofilin | AR, LoF | Moderate neutropenia, migration defect, abnormal spreading,  increased basal F-actin levels, enhanced ROS production, and abnormal location of the nucleus | Severe stomatitis, recurrent infections and fever | [20-22] |
| **DOCK2** | GEF activating RAC | AR, LoF | T-cell lymphopenia, impaired actin polymerization and impaired  ROS production | Severe bacterial and viral infections | [23] |
| **DOCK8** | GEF activating CDC42 | AR, LoF | T-cell lymphopenia, mild motility defect | Severe recurrent viral, bacterial, and fungal infections, eczema, and allergies | [24] |
| **DOCK11** | Activates CDC42 | XR, LoF | T cells cannot form filopodia and showed abnormal migration, platelets exhibited abnormal ultrastructural morphology and spreading, abnormal actin cytoskeleton remodeling | Infections, early-onset severe immune dysregulation, normocytic anemia of variable severity associated with anisopoikilocytosis, autoimmunity and developmental delay | [25] |
| **RAC2** | Rho GTPase involved in the respiratory burst and  activator of the WRC | AR, LoF | Lymphopenia, decreased F-actin and impaired actin polymerization | Recurrent infections, hypogammaglobulinemia | [26,27] |
|  |  | AD, LoF | Neutrophilia, impaired chemotactic response, decreased basal  F-actin, impaired actin polymerization, decreased ROS production | Severe bacterial infections, poor wound-healing | [28] |
|  |  | AD, GoF | Neutropenia, lymphopenia, impaired chemotaxis, increased F-actin levels and actin polymerization, increased ROS and apoptosis | Recurrent respiratory infections | [29,30] |
| **Moesin** | Links actin filaments to the plasma membrane and  as a signaling mediator | XR, LoF | Lymphopenia, neutropenia, monocytopenia, thrombocytopenia. | Recurrent bacterial and viral infections, recurrent molluscum, eczema | [31] |
| **STK4** | Kinase for nuclear proteins and activator of RAC | AR, LoF | T-lymphopenia, neutropenia, loss of mitochondrial membrane potential and susceptibility to apoptosis | Recurrent bacterial and viral infections, mucocutaneous candidiasis, eczema, molluscum contagiosum, high IgA/E  levels, cardiac aberrations | [32] |
| **LCP1** | Actin-binding protein | AD, GoF | Severe neutropenia, lymphopenia, impaired cell migration, increased F-actin levels and actin polymerization, mitochondrial dysfunction and increased apoptosis, cell cycle arrest | Recurrent bacterial and viral infections, eczema, urticaria, poor wound healing | This report; [33,34] |
|  |  | AD, LoF | Lymphopenia, neutropenia, and thrombocytopenia, Dominant negative action of altered LPL results in defects in lymphocyte migration and cytokinesis | Recurrent infections, including fevers, recurrent otitis media, and tonsillitis. | [35] |

LoF, Loss-of-funtion. GoF, Gain-of-funtion. AR, autosomal recessive inheritance. AD, autosomal dominant inheritance. XR, X-linked recessive inheritance.

**Supplementary references**

1. Yu L, Li W, Lv G, Sun G, Yang L, Chen J, Zhou L, Ding Y, Zhang Z, Tang X, An Y, Zhao X. De Novo Somatic Mosaicism of CYBB Caused by Intronic LINE-1 Element Insertion Resulting in Chronic Granulomatous Disease. J Clin Immunol. 2023 Jan;43(1):88-100. doi: 10.1007/s10875-022-01347-w
2. Bylund J, Björnsdottir H, Sundqvist M, Karlsson A, Dahlgren C. Measurement of respiratory burst products, released or retained, during activation of professional phagocytes. Methods Mol Biol. 2014;1124:321-38. doi: 10.1007/978-1-62703-845-4_21.
3. Li W, Sun Y, Yu L, Chen R, Gan R, Qiu L, Sun G, Chen J, Zhou L, Ding Y, Du H, Shu Z, Zhang Z, Tang X, Chen Y, Zhao X, Zhao Q, An Y. Multiple Immune Defects in Two Patients with Novel DOCK2 Mutations Result in Recurrent Multiple Infection Including Live Attenuated Virus Vaccine. J Clin Immunol. 2023 Aug;43(6):1193-1207. doi: 10.1007/s10875-023-01466-y.
4. Lionakis MS, Lim JK, Lee CC, Murphy PM. Organ-specific innate immune responses in a mouse model of invasive candidiasis. J Innate Immun. 2011;3(2):180-99. doi: 10.1159/000321157.
5. Swamydas M, Lionakis MS. Isolation, purification and labeling of mouse bone marrow neutrophils for functional studies and adoptive transfer experiments. J Vis Exp. 2013 Jul 10;(77):e50586. doi: 10.3791/50586.
6. Hou L, Voit RA, Shibamura-Fujiogi M, Koutsogiannaki S, Li Y, Chen Y, Luo H, Sankaran VG, Yuki K. CD11c regulates neutrophil maturation. Blood Adv. 2023 Apr 11;7(7):1312-1325. doi: 10.1182/bloodadvances.2022007719.
7. Evrard M, Kwok IWH, Chong SZ, Teng KWW, Becht E, Chen J, Sieow JL, Penny HL, Ching GC, Devi S, Adrover JM, Li JLY, Liong KH, Tan L, Poon Z, Foo S, Chua JW, Su IH, Balabanian K, Bachelerie F, Biswas SK, Larbi A, Hwang WYK, Madan V, Koeffler HP, Wong SC, Newell EW, Hidalgo A, Ginhoux F, Ng LG. Developmental Analysis of Bone Marrow Neutrophils Reveals Populations Specialized in Expansion, Trafficking, and Effector Functions. Immunity. 2018 Feb 20;48(2):364-379.e8. doi: 10.1016/j.immuni.2018.02.002.
8. Nunoi H, Yamazaki T, Tsuchiya H, et al. A heterozygous mutation of beta-actin associated with neutrophil dysfunction and recurrent infection. *Proc Natl Acad Sci U S A*. 1999;96(15):8693-8698. doi:10.1073/pnas.96.15.8693
9. Derry JM, Ochs HD, Francke U. Isolation of a novel gene mutated in Wiskott-Aldrich syndrome [published correction appears in Cell. 1994 Dec 2;79(5):following 922]. *Cell*. 1994;78(4):635-644. doi:10.1016/0092-8674(94)90528-2
10. Devriendt K, Kim AS, Mathijs G, et al. Constitutively activating mutation in WASP causes X-linked severe congenital neutropenia. *Nat Genet*. 2001;27(3):313-317. doi:10.1038/85886
11. Keszei M, Record J, Kritikou JS, et al. Constitutive activation of WASp in X-linked neutropenia renders neutrophils hyperactive [published correction appears in J Clin Invest. 2021 Dec 1;131(23):]. *J Clin Invest*. 2018;128(9):4115-4131. doi:10.1172/JCI64772
12. Record J, Malinova D, Zenner HL, et al. Immunodeficiency and severe susceptibility to bacterial infection associated with a loss-of-function homozygous mutation of MKL1. *Blood*. 2015;126(13):1527-1535. doi:10.1182/blood-2014-12-611012
13. Sprenkeler EGG, Henriet SSV, Tool ATJ, et al. MKL1 deficiency results in a severe neutrophil motility defect due to impaired actin polymerization. *Blood*. 2020;135(24):2171-2181. doi:10.1182/blood.2019002633
14. Kuijpers TW, Tool ATJ, van der Bijl I, et al. Combined immunodeficiency with severe inflammation and allergy caused by ARPC1B deficiency. *J Allergy Clin Immunol*. 2017;140(1):273-277.e10. doi:10.1016/j.jaci.2016.09.061
15. Nunes-Santos CJ, Kuehn H, Boast B, et al. Inherited ARPC5 mutations cause an actinopathy impairing cell motility and disrupting cytokine signaling. *Nat Commun*. 2023;14(1):3708. Published 2023 Jun 22. doi:10.1038/s41467-023-39272-0
16. Castro CN, Rosenzwajg M, Carapito R, et al. NCKAP1L defects lead to a novel syndrome combining immunodeficiency, lymphoproliferation, and hyperinflammation. *J Exp Med*. 2020;217(12):e20192275. doi:10.1084/jem.20192275
17. Cook SA, Comrie WA, Poli MC, et al. HEM1 deficiency disrupts mTORC2 and F-actin control in inherited immunodysregulatory disease. *Science*. 2020;369(6500):202-207. doi:10.1126/science.aay5663
18. Lanzi G, Moratto D, Vairo D, et al. A novel primary human immunodeficiency due to deficiency in the WASP-interacting protein WIP. *J Exp Med*. 2012;209(1):29-34. doi:10.1084/jem.20110896
19. Yee CS, Massaad MJ, Bainter W, et al. Recurrent viral infections associated with a homozygous CORO1A mutation that disrupts oligomerization and cytoskeletal association. *J Allergy Clin Immunol*. 2016;137(3):879-88.e2. doi:10.1016/j.jaci.2015.08.020
20. Standing AS, Malinova D, Hong Y, et al. Autoinflammatory periodic fever, immunodeficiency, and thrombocytopenia (PFIT) caused by mutation in actin-regulatory gene WDR1. *J Exp Med*. 2017;214(1):59-71. doi:10.1084/jem.20161228
21. Pfajfer L, Mair NK, Jiménez-Heredia R, et al. Mutations affecting the actin regulator WD repeat-containing protein 1 lead to aberrant lymphoid immunity. *J Allergy Clin Immunol*. 2018;142(5):1589-1604.e11. doi:10.1016/j.jaci.2018.04.023
22. Kuhns DB, Fink DL, Choi U, et al. Cytoskeletal abnormalities and neutrophil dysfunction in WDR1 deficiency. *Blood*. 2016;128(17):2135-2143. doi:10.1182/blood-2016-03-706028
23. Dobbs K, Domínguez Conde C, Zhang SY, et al. Inherited DOCK2 Deficiency in Patients with Early-Onset Invasive Infections. *N Engl J Med*. 2015;372(25):2409-2422. doi:10.1056/NEJMoa1413462
24. Engelhardt KR, McGhee S, Winkler S, et al. Large deletions and point mutations involving the dedicator of cytokinesis 8 (DOCK8) in the autosomal-recessive form of hyper-IgE syndrome [published correction appears in J Allergy Clin Immunol. 2010 Mar;125(3):743. Kutuculer, Necil [corrected to Kutukculer, Necil]]. *J Allergy Clin Immunol*. 2009;124(6):1289-302.e4. doi:10.1016/j.jaci.2009.10.038
25. Block J, Rashkova C, Castanon I, et al. Systemic Inflammation and Normocytic Anemia in DOCK11 Deficiency. *N Engl J Med*. 2023;389(6):527-539. doi:10.1056/NEJMoa2210054
26. Ambruso DR, Knall C, Abell AN, et al. Human neutrophil immunodeficiency syndrome is associated with an inhibitory Rac2 mutation. *Proc Natl Acad Sci U S A*. 2000;97(9):4654-4659. doi:10.1073/pnas.080074897
27. Williams DA, Tao W, Yang F, et al. Dominant negative mutation of the hematopoietic-specific Rho GTPase, Rac2, is associated with a human phagocyte immunodeficiency. *Blood*. 2000;96(5):1646-1654.
28. Alkhairy OK, Rezaei N, Graham RR, et al. RAC2 loss-of-function mutation in 2 siblings with characteristics of common variable immunodeficiency. *J Allergy Clin Immunol*. 2015;135(5):1380-4.e45. doi:10.1016/j.jaci.2014.10.039
29. Hsu AP, Donkó A, Arrington ME, et al. Dominant activating RAC2 mutation with lymphopenia, immunodeficiency, and cytoskeletal defects. *Blood*. 2019;133(18):1977-1988. doi:10.1182/blood-2018-11-886028
30. Zhang L, Chen Z, Li W, et al. Combined Immunodeficiency Caused by a Novel De Novo Gain-of-Function RAC2 Mutation. *J Clin Immunol*. 2022;42(6):1280-1292. doi:10.1007/s10875-022-01288-4
31. Lagresle-Peyrou C, Luce S, Ouchani F, et al. X-linked primary immunodeficiency associated with hemizygous mutations in the moesin (MSN) gene. *J Allergy Clin Immunol*. 2016;138(6):1681-1689.e8. doi:10.1016/j.jaci.2016.04.032
32. Abdollahpour H, Appaswamy G, Kotlarz D, et al. The phenotype of human STK4 deficiency. *Blood*. 2012;119(15):3450-3457. doi:10.1182/blood-2011-09-378158
33. Mahat U, Garg B, Yang CY, et al. Lymphocyte cytosolic protein 1 (L-plastin) I232F mutation impairs granulocytic proliferation and causes neutropenia. Blood Adv. 2022;6(8):2581-2594. doi:10.1182/bloodadvances.2021006398
34. Marti S, Pellet P, Beaupain B, et al. Expanding the phenotypic and genetic landscape of congenital neutropenia through whole-exome and genome sequencing. Hemasphere. 2025;9(6):e70150. Published 2025 Jun 11. doi:10.1002/hem3.70150
35. Hernandez RA, Hearn JI, Bhoopalan V, Hamzeh AR, Kwong K, Diamand K, Davies A, Li FJ, Padmanabhan H, Milne R, Ballard F, Spensberger D, Gardiner EE, Miraghazadeh B, Enders A, Cook MC. L-plastin associated syndrome of immune deficiency and hematologic cytopenia. J Allergy Clin Immunol. 2024 Sep;154(3):767-777. doi: 10.1016/j.jaci.2024.05.001.
36. Jia Y, Yang Q, Wang Y, et al. Hyperactive PI3Kδ predisposes naive T cells to activation via aerobic glycolysis programs. Cell Mol Immunol. 2021;18(7):1783-1797. doi:10.1038/s41423-020-0379-x
37. Bainter W, Platt CD, Park SY, et al. Combined immunodeficiency due to a mutation in the γ1 subunit of the coat protein I complex. J Clin Invest. 2021;131(3):e140494. doi:10.1172/JCI140494
38. van Bergen T, Bosch D, Bellanné-Chantelot C, Mulet-Lazaro R, Bledsoe JR, Chen L, de Looper HWJ, van Dijk C, Ter Borg M, Bindels E, Hoogenboezem R, Roovers O, Olofsen PA, Bartels M, van Montfrans JM, Breed P, Salzer E, Holierhoek MG, Nelken B, Beaupain B, Fleming MD, Shimamura A, Raaijmakers MHGP, Donadieu J, Newburger PE, Touw IP, Aalbers AM. Germline LCP1 mutations cause immunodeficiency with neutropenia, monocytopenia, lymphopenia and defective cytokinesis. Blood Adv. 2025 Oct 7:bloodadvances.2025016507. doi: 10.1182/bloodadvances.2025016507.

**Supplementary Figures**

**Supplementary Figure 1.** Peripheral blood cell phenotypes of three patients with neutropenia and immunodeficiency. (A) The bone marrow aspirate smear of P3 shows giant tetraploid karyotype neutrophils. (Wright’s stain, magnification × 1000).

**Supplementary Figure 2.** Identification of L362F-iPSC induced from PBMC of P1. (A) The expression of L362F-iPSC pluripotent markers (OCT4, SOX2, TRA-1-60 and SSEA4) was verified positive by immunofluorescence staining. (B) In vivo teratoma formation demonstrated the ability of L362F-iPSC to differentiate into three germ layers. (C) Sanger sequencing confirmed that L362F-iPSC had a heterozygous mutation of c.1086G>C. (D) (Short Tandem Repeat) STR analysis confirmed that all STR loci of L362F-iPSC were identical to P1. (E) Comparable granulocyte clones size of HC and L362F-iPSC were analyzed on days 12-14 of induced neutrophil maturation. (F) Sanger sequencing of LCP1 mutation sites in gene-edited iPSCs. (G) Representative Wright's-Giemsa staining cytospin preparation of gene-edited iPSCs-derived neutrophils on the day 12-14. The original magnification used a 100 × objective. (H)-(J) Quantification of mitochondrial parameters in iPSCs-derived neutrophils. (H) MTG. (I) Mitochondrial ROS (mtROS). (J) MMP per mitochondrion (TMRM/MTG) .

**Supplementary Figure 3.** T cell and NK cell immunological phenotypes in patients with LCP1 mutations. (A) and (B) Flow cytometry showed increased expression of CD57,HLA-DR, and PD-1 in CD4^+^ and CD8^+^ T lymphocytes associated with aging in P2. (C) Representative flow cytometry showed the percentage of CXCR5^+^CD45RO^+^ Tfh cells in CD4^+^ T cells. (D) Expression of PD-1 and ICOS in CD4^+^CXCR5^+^CD45RO^+^ Tfh cells. (E) CXCR5^+^CD45RA^-^CD127^−^CD25^+^ Tfr cells gating on CD4^+^ T cells. (F) FOXP3^+^CD25^+^ Treg cells gating on CD4^+^ T cells. (G) Th1, Th2 and Th17 cells in CXCR5^-^CD45RA^-^ cells and the distribution of Th1-like, Th2-like and Th17-like cells in CXCR5^+^CD45RA^-^ cells. (H) CD107a surface expression of CD3^-^CD56^+^ NK cells treated with K562. (I) Expression of mature molecules CD25, CD69, and ICOS on the surface of P2’s T cells in the presence of CD3 or CD3/28. (J) and (K) Normal production of IL-2, IL-4, IFN-γand IL-17A in P2 after 72h stimulation by PMA and ionomycin. (L) Western blot showed early normal TCR signaling in P1 stimulated by CD3/28.

**Supplementary Figure 4.** Over-polymerized F-actin in patients' primary lymphocytes, neutrophils, and the protein stability of overexpression of LCP1 L362F and A365D. (A) - (D) Flow cytometry showed the expression of F-actin and LCP1 in T and B lymphocytes and neutrophils of P1 and P2. (E) The protein stability of overexpression of LCP1 L362F and A365D in 293T cells was detected by cycloheximide chase assay.

**Supplementary Figure 5.** Dysfunctional mitochondrion organization and cell cycle in LCP1 mutant patients. (A) Expression of cell type specific markers in the defined clusters. (B) Flow cytometry analysis of bone marrow samples from Patient P1 was performed using CD11b and CD16 expression to distinguish between mature and immature neutrophil populations. (C) The volcano plot shows the differentially expressed genes in each cluster between P1 and HCs , statistically significant actin filament organization related genes were colored as red (up-regulated in P1). (D) Heatmap shows the foldchanges of top 6 cytoskeleton related proteins related genes with at least 1.5 foldchanges in each cluster. (E) Gene set enrichment analysis plot of upregulated DEGs in P1 proNeu cells enriched in mitochondrion-related pathway, P = 0.06. (F) Heatmap shows the foldchanges of top 6 mitochondrion organization related genes with at least 1.5 foldchanges in each cluster. (G) Mitochondrial membrane potential was measured with Tetramethylrhodamine (TMRM) by flow cytometry for P1 and P2. (H) Cell cycle of neutophils was analyzed with flow cytometry. (I) Heatmap shows the foldchanges of top 6 apoptosis related genes with at least 1.5 foldchanges in each cluster. (J-L) The bubble chart shows the functional analysis of differentially expressed genes in mature B cells (J), pro-B cells (K), and pre-B cells (L). (M) QPCR analysis of the relative expression of selected genes associated with cell stress. Each experiment was conducted at least three times.

**Supplementary Figure 6.** Immunological phenotypic identification of LCP1^+/L362F^ mice. (A) Sanger sequencing confirmed the mice genotypes of HE and HO. (B) Flow cytometry analysis for peripheral blood neutrophils and lymphocytes in mice (n>3). (C) Number of viable bone marrow cells, thymocytes, spleen and lymph node (LNs) cells. (D) Wright's-Giemsa composite staining of bone marrow neutrophils from WT and HE, micemagnification ×1000. The black arrow indicates mature neutrophils, while the red arrow indicates immature neutrophils and neutrophil precursor cells. (E) Electron microscopy images of neutrophils in the bone marrow of LCP1^+/L362F^ mice. (F) Normal phosphorylation levels of LCP1 and Ser5 in bone marrow neutrophils of LCP1^+/L362F^ mice. (G) Distribution of thymocyte subsets in LCP1^+/L362F^ mice and WT controls. (H) Number of viable of splenic T (CD3^+^) cells, B (B220^+^) cells, iNKT (TCRb^+^CD1d^+^) cells, innate lymphoid cells (CD3^-^ LinCD90.2^+^) and CD3- NK1.1^+^ NK cells in mutant mice and WT controls. (I) Percentages of spleen and thymus CD4^+^FOXP3^+^ total Tregs in mutant mice and WT controls. (J) Distribution of CD4^+^ and CD8^+^ T cells among CD3^+^ cells in spleen, inguinal lymph nodes (ILN), and mesenteric lymph node (MLN), percentages of splenic naive and memory CD4^+^ cells and CD8^+^ cells. (K) Proliferation of CD4^+^ T cells, CD8^+^ T cells, and CD19^+^ B cells in LCP1^+/L362F^ versus WT mice. (L) Surface expression of CD25 and CD69 on magnetic bead-sorted CD4+ T cells after 2-day stimulation with anti-CD3/CD28 antibodies. (M) Magnetic bead-sorted CD4+ T cells were treated with Golgi-blocking reagents after 3 days of anti-CD3/CD28 stimulation for intracellular cytokine analysis (IFN-γ, IL-2, TNF-α). (N) Analysis of lymphocyte senescence markers (KLRG1 and T-bet) in young (2-month-old) versus aged (14-month-old) mice. (O) The distribution of splenic B cell subsets, including T1 (CD21^high^CD24^low^), T2 (CD21^high^CD24^int^), follicular mature (FM) (CD21^int^CD24^low^), marginal zone (MZ) (CD21^int^CD24^high^CD23^low^) and marginal zone precursor (MZP) (CD21^int^CD24^high^CD23^high^) B cells. (P) The development of bone marrow B cells. Left: gating strategy of pro-B, pre-B, late-pre B, immature B and recirculating B cells. Right: quantification.

**Supplementary Figure 7.** Phenotypes and circular gating strategy of peripheral blood and bone marrow cells in LCP1^+/L362F^ mice. (A) Flow cytometry analysis for mice bone marrow neutrophils (B220^-^F4/80^-^CD11b^+^Ly6G^+^) and mononuclear-macrophages (B220^-^F4/80^+^CD11C^+/low^Ly6G^+^). (B) Circular gating strategy of CMPs (CD16/32^low^CD34^+^) and GMPs (CD16/32^high^CD34^+^). (C) Circular gating strategy of proNeu (Lin^-^Gr-1^+^CD11b^+^CXCR4^+^c-kit^int^CXCR2^-^), immature neutrophil (Lin^-^Gr-1^+^CD11b^+^CXCR4^-/low^c-kit^low/-^CXCR2^-^), mature neutrophil (Lin^-^Gr-1^+^CD11b^+^CXCR4^-/low^c-kit^low/-^CXCR2^+^) in LCP1^+/L362F^ mice bone marrow (n>6).

**Supplementary Figure 8.** Over-polymerized F-actin in LCP1^+/L362F^ mice. (A) Confocal microscopy analysis of LCP1^+/L362F^ patient-derived neutrophils, co-stained for F-actin (phalloidin, green), VDAC1 (red), and the mitochondrial marker Tomm20 (pink). (B) The inhibitory effects of Latrunculin B (LatB) and Oroxylin A on F-actin polymerization.
